# Supplementary material for: Directionality of developing skeletal muscles is set by mechanical forces
Source: Nat Commun. 2023 May 27;14:3060. doi: 10.1038/s41467-023-38647-7 (PMC10224984; doi:10.1038/s41467-023-38647-7)
Supplement: Supplementary file 1 — Supplementary Information [file 41467_2023_38647_MOESM1_ESM.pdf]

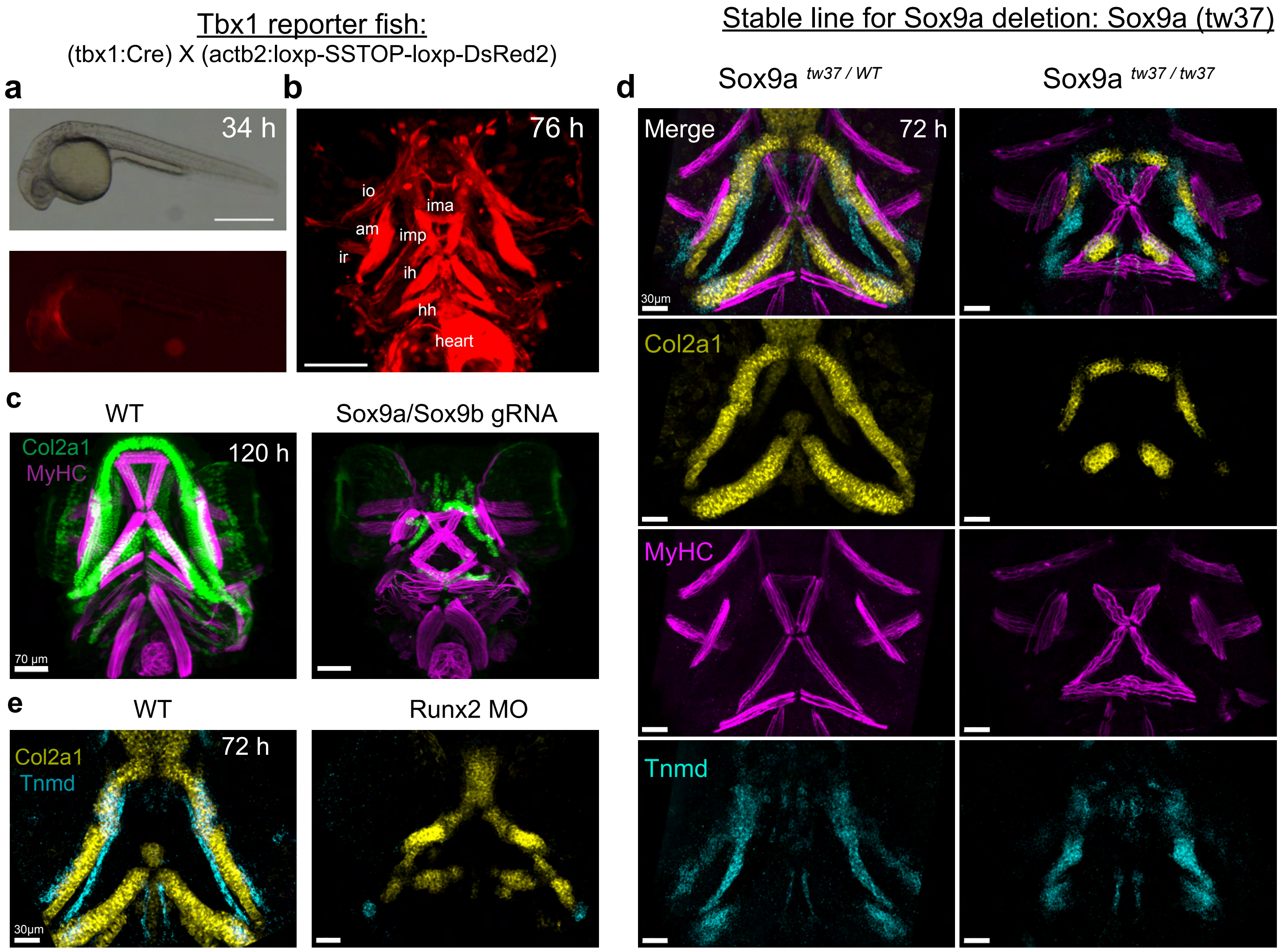

Supplementary Figure 1

Supplementary Figure 1. A transgenic reporter for tracking muscle differentiation in zebrafish.

(a) Embryos from an outcross between Tg(*tbx1:Cre*) and Tg(*actb2:loxp:sstop:loxp-DsRed*) were visualized with bright-field microscopy (upper panel) or fluorescent microscopy for DsRed (lower panel). **(b)** The facial muscle of the embryo from the outcross between Tg(*tbx1:Cre*) and Tg(*actb2:loxp:sstop:loxp-DsRed*) is shown. Abbreviation; io, inferior oblique. ir, inferior rectus. imp, inermandibularis posterior. ima, intermandibularis anterior. am, adductor mandibulae. ih, interhyoideus. hh, hyohyoideus.

(c) Control and Sox9-perturbed embryos at 120 hpf stained for Col2a1:mCherry and MyHC.

(d) Sox9a^{tw37} heterozygous (left) and homozygous (right) embryos at 72 hpf analyzed for gene expression using HCR for Col2a1 and Tenomodulin (Tnmd), and thereafter immunostained for MyHC.

(e) Runx2 MO-injected and control embryo at 72 hpf analyzed for gene expression using HCR for Col2a1 and Tenomodulin.

Scale bar; (a), 500 μm. (b), 100 μm. (c), 70 μm. (d) and (e), 30 μm.

Timeline of myocyte and tenocyte differentiation in zebrafish and mouse

a

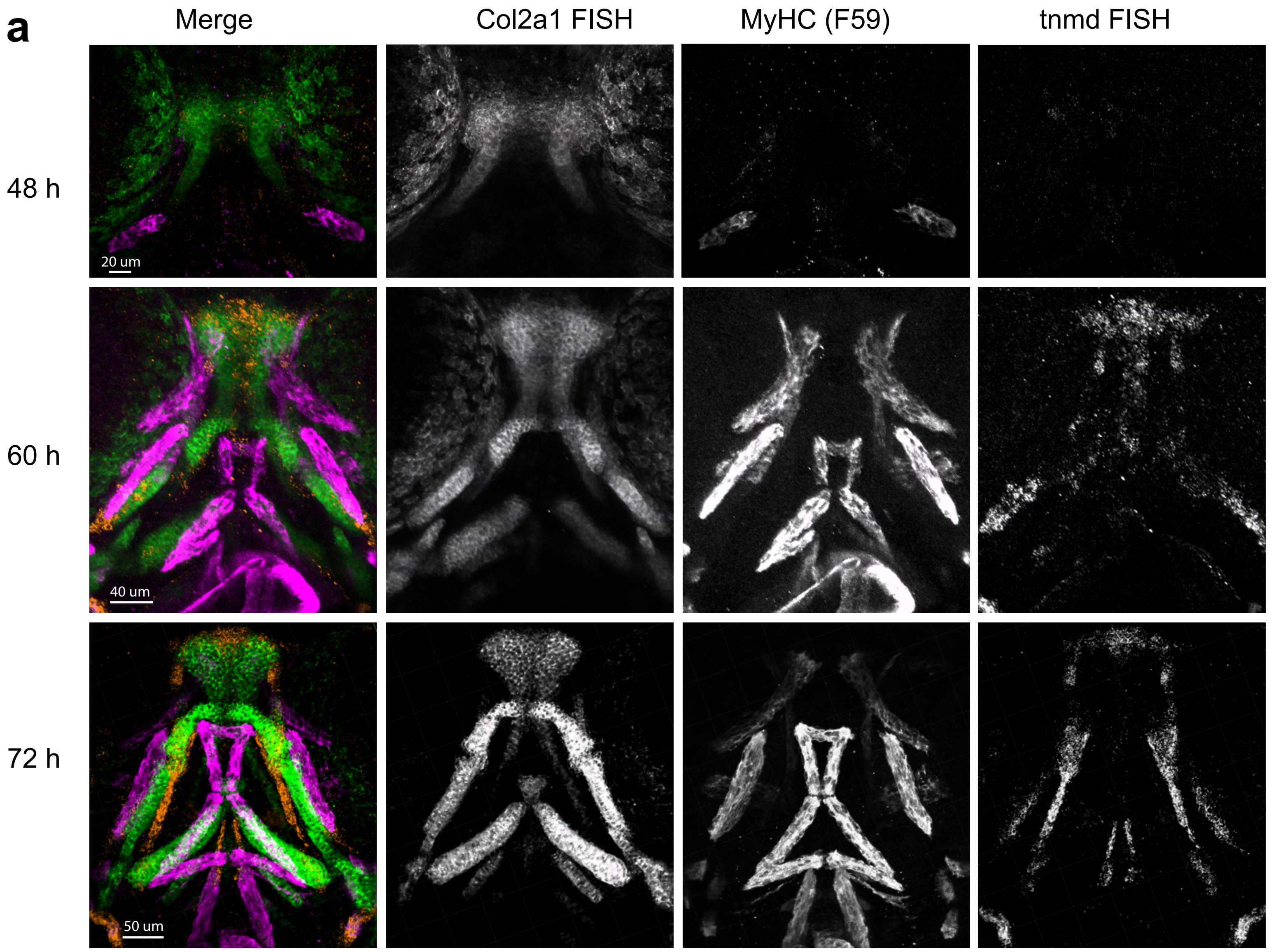

Genetic perturbations of tenocyte ECM proteins do not affect myocyte polarization

b

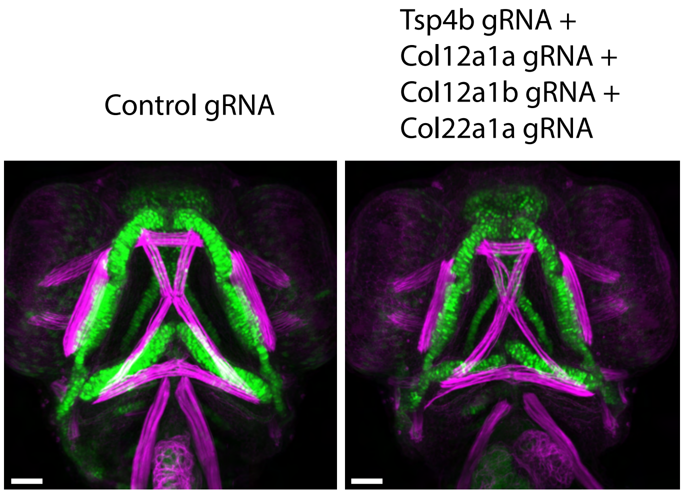

c

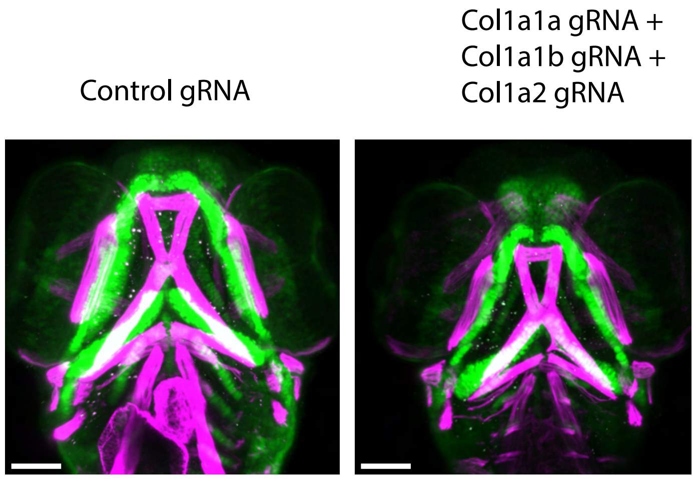

Supplementary Figure 2

Supplementary Figure 2. Mature tenocytes are not required for myocyte polarization in zebrafish embryos.

(a) Timeline of cartilage, muscle, and tendon co-development in zebrafish embryo via snapshots at 48, 60, and 72 hpf. Fish were immunostained with MyHC (F59) after HCR-based in situ hybridization for analyzing gene expression of Col2a1 and Tnmd.

(b) Control-gRNA treated embryos, and embryos perturbed with gRNA pools targeting the components of the tenocyte extracellular matrix Tsp4b, Col12a1a, Col12a1b, Col22a1a, stained for Col2a1:mCherry and MyHC

(c) Control-gRNA treated embryos, and embryos perturbed with gRNA pools targeting the components of the tenocyte extracellular matrix Col1a1a, Col1a1b, Col1a2, stained for Col2a1:mCherry and MyHC.

Scale bars; in (a) top, 20 μm, middle, 40 μm, bottom, 50 μm. (b), 50 μm. (c), 70 μm.

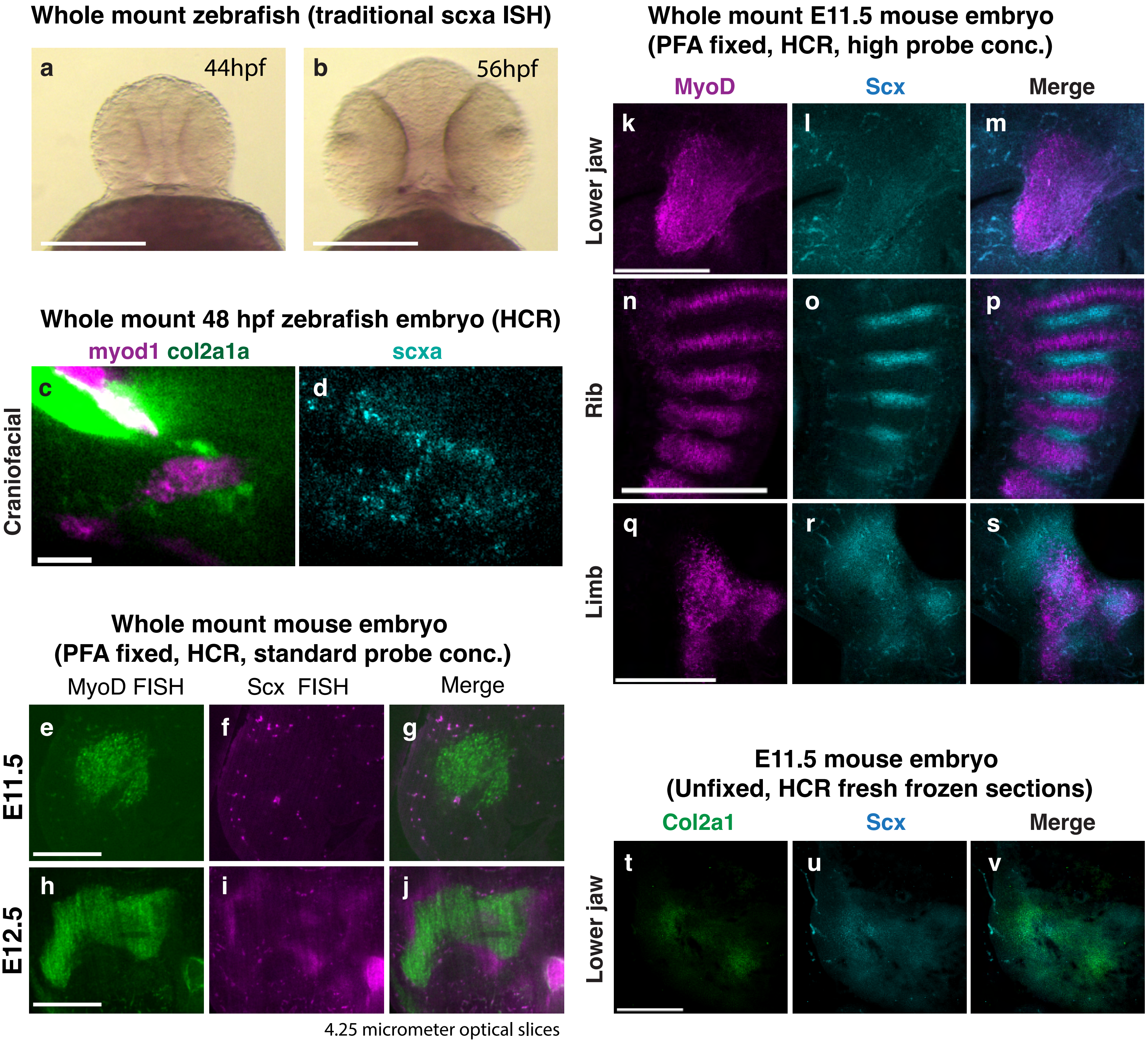

Supplementary Figure 3

Supplementary Figure 3. Detecting Scleraxis expression in the zebrafish and mouse embryonic connective tissue primordia.

(a-b) Whole mount traditional *in situ* hybridization for Scleraxis (*scxa*) in zebrafish embryos at 44 hpf (a) and 56 hpf (b). Dark brown regions indicate areas of *scxa* expression.

(c-d) Whole mount HCR-based *in situ* hybridization using standard probe concentrations to compare myocyte (*myod1*, magenta) and chondrocyte (*col2a1a*, green) gene expression (c), versus tenocyte precursor (*scxa*, cyan) gene expression (d) in PFA-fixed zebrafish craniofacial mesenchyme at 48 hpf.

(e-j) Whole mount HCR-based *in situ* hybridization using standard probe concentrations to compare myocyte (*MyoD* in green, e and h) and tenocyte (*Scx* in magenta, f and i) gene expression in PFA-fixed mouse facial mesenchyme at embryonic days 11.5 (e-g) and 12.5 (h-j). Optical slices are shown (thickness 4.25 µm).

(k-s) Whole mount HCR-based *in situ* hybridization using 2X probe concentrations to compare myocyte (*MyoD* in magenta, k, n, q) and tenocyte precursor (*Scx* in cyan, l, o, r) in PFA-fixed mouse embryos at different anatomical regions such as lower jaw (k-m), ribs (n-p), and limb (q-s) at embryonic day 11.5.

(t-v) HCR-based *in situ* hybridization using standard probe concentrations on unfixed, fresh frozen sections to compare chondrocyte (*Col2a1* in green, t) versus tenocyte precursor (*Scx*, cyan, u) in 11.5 mouse embryonic lower jaw tissue.

Scale bars; in (a-b), 200 µm. (c-d), 50 µm. (e-j), 300 µm. (k-v), 500 µm.

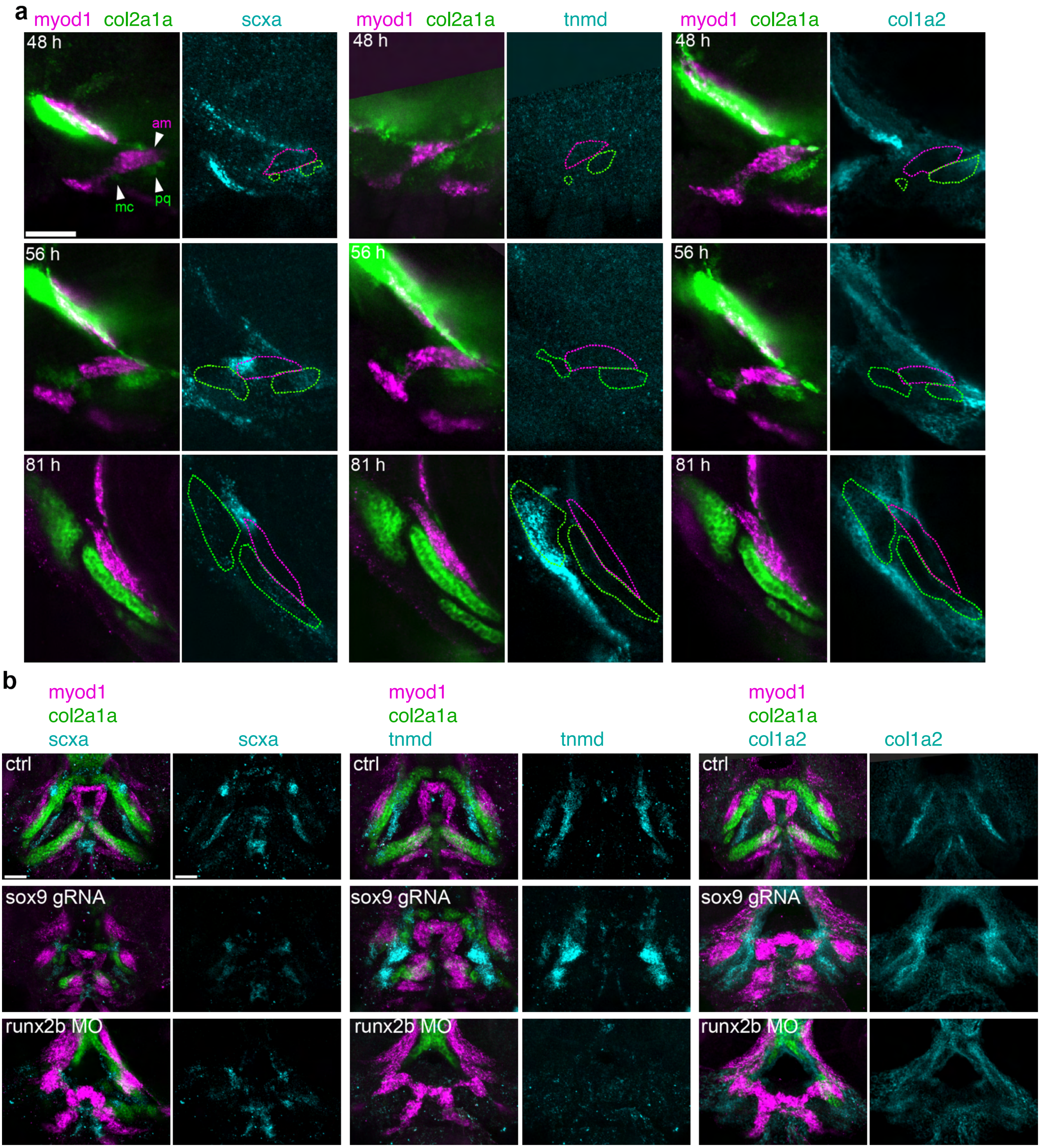

Supplementary Figure 4

Supplementary Figure 4. Developmental regulation of tenocyte gene expression.

(a) Time course experiment where gene expression is assessed via hybridization chain reaction (HCR) using probes specific to tenocyte markers: either Scleraxis (*scxa*), Tenomodulin (*tnmd*), or Collagen 2 isoform a1 (*col1a2*) in conjunction with cartilage marker *col2a1a* and muscle marker *myod1*. Dotted lines outline the muscle or cartilage in the indicated channel. Embryos were harvested for gene expression analysis at either 48, 56, or 81 hpf. Abbreviations: am, adductor mandibulae; mc, Meckel's cartilage; pq, paloquadrate.

(b) Gene expression is assessed via HCR for the indicated combinations of marker genes at 81 hpf for the following conditions: top row, control gRNA injected; middle row, sox9a+sox9b gRNA injected; bottom row, Runx2b-MO injected.

Images are representative of n=2-3 fish per condition.

Scale bars; 50 μm.

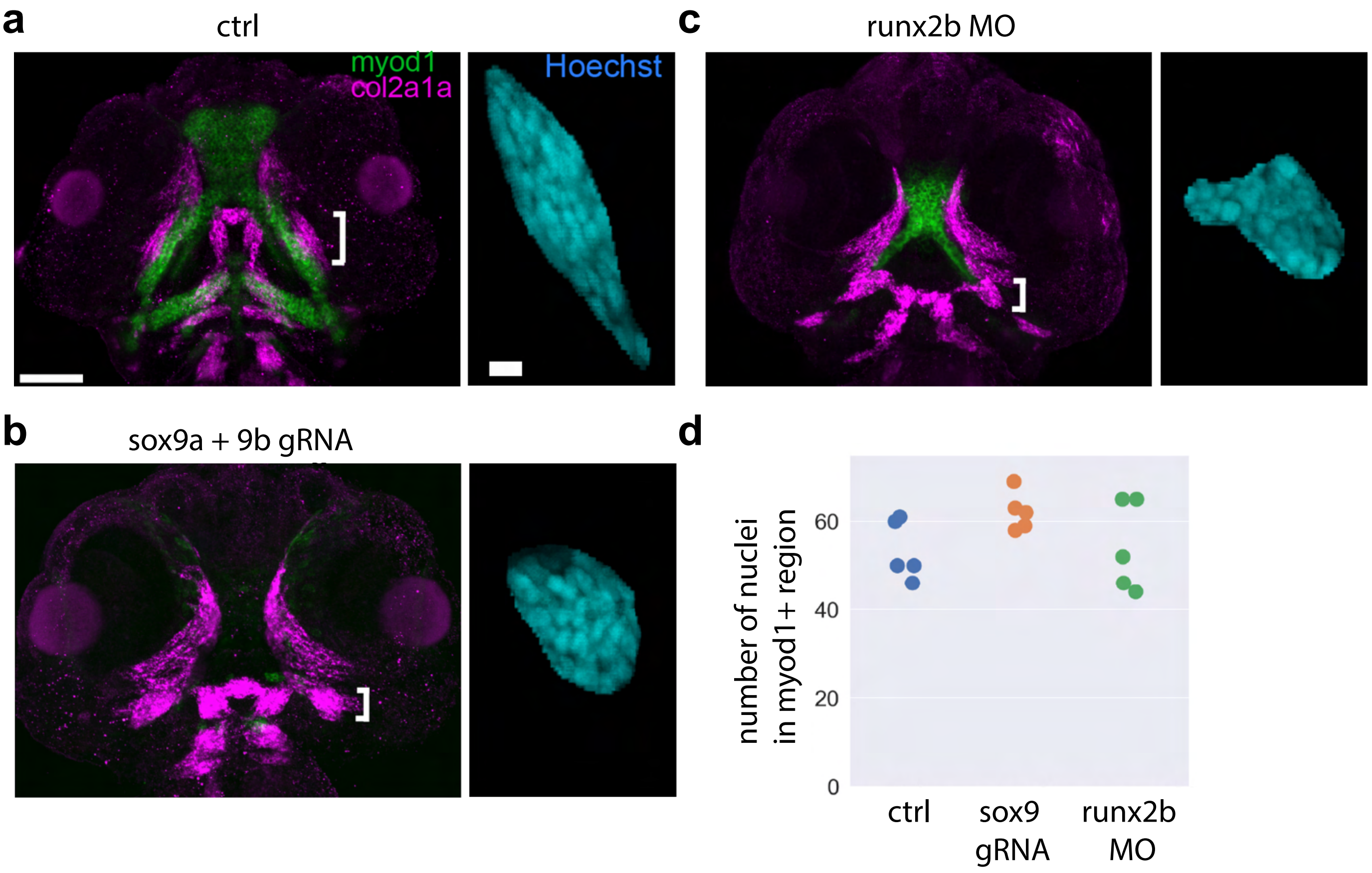

Supplementary Figure 5

Supplementary Figure 5. Cartilage ablation does not affect the number of cells per muscle.

(a-d) 81 hpf zebrafish embryos analyzed with HCR for *myod1*, *col2a1a*, and nuclei distinguished using Hoechst labeling. Top left, control gRNA-injected embryos (a); top right, Runx2b-MO injected embryos (b); bottom left, sox9a+sox9b gRNA-injected embryos (c). Bottom right (d), quantification of nuclei found per muscle (defined as *myod1*$^{+}$ region). Square brackets indicate location of inset. Scale bar; left, 100 μm. right, 10 μm.

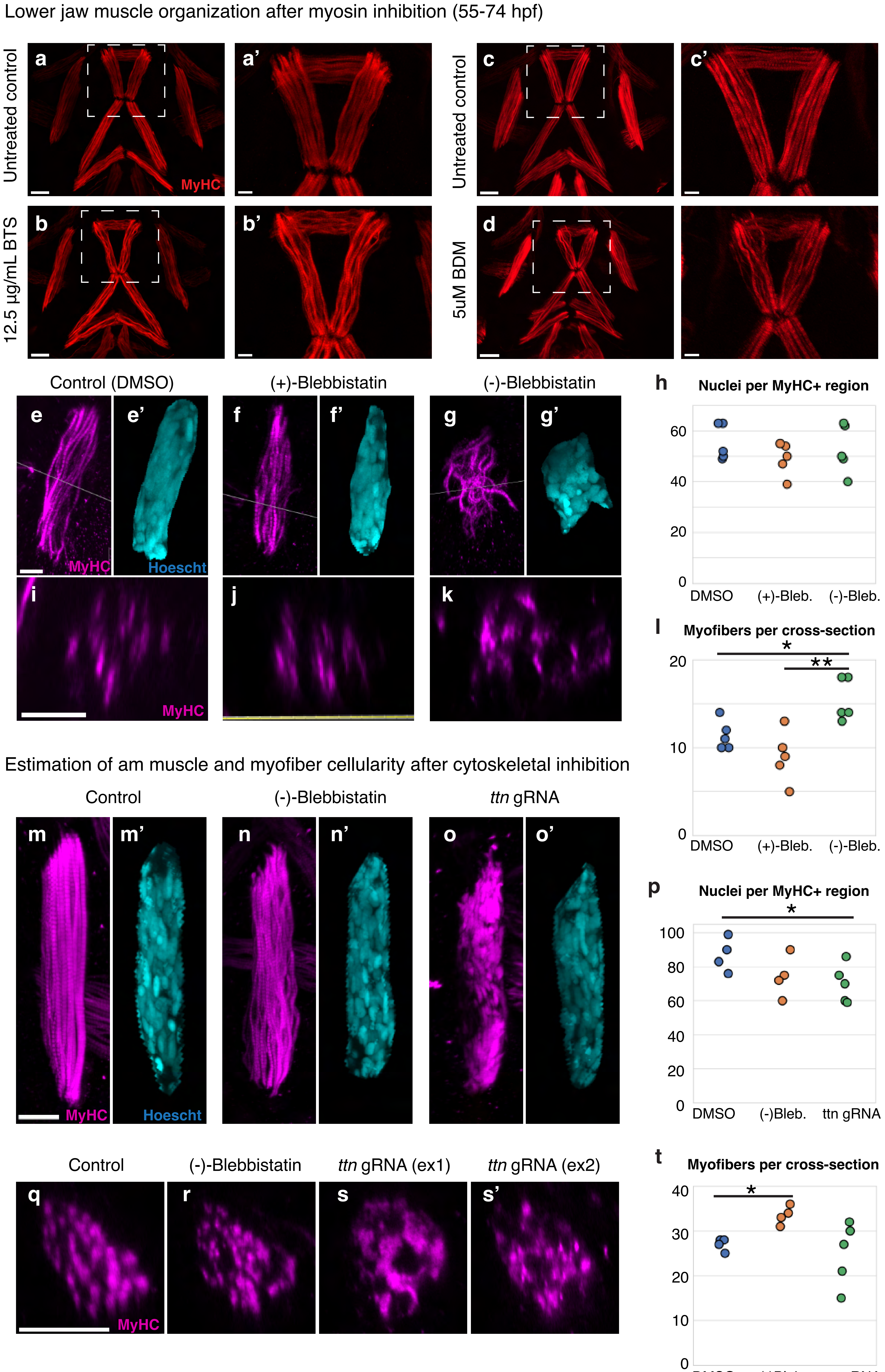

Supplementary Figure 6

Supplementary Figure 6. Effects of cytoskeletal inhibition on myofiber development.

(a-b) Zebrafish embryos were either untreated (a) treated with 12.5 µg/mL BTS (b) from 55 hpf and muscle organization was analyzed by MyHC staining at 74 hpf. The dotted white box is magnified and shown beside the overview in (a') and (b').

(c-d) Zebrafish embryos were either untreated (c) treated with 5 µM BDM (d) from 55 hpf and muscle organization was analyzed by MyHC staining at 74 hpf. The dotted white box is magnified and shown beside the overview in (c') and (d').

(e-h) Zebrafish embryos were either exposed to DMSO vehicle control (e), inactive enantiomer (+)-Blebbistatin (f), or active enantiomer (-)-Blebbistatin (g) from 39 hpf and stained for MyHC and Hoescht to analyze myofiber organization and nuclear amounts in the AM muscle at 52 hpf. The MyHC-positive region was segmented to reveal muscle cell nuclei in 3D (e', f', g'). Nuclei count quantifications are shown in (h). Statistically significant differences were not found (unpaired two-tailed Student's t-test).

(i-l) A cross-sectional image through the vehicle control (i), inactive control (j), and active Blebbistatin (k) is shown at the level indicated by the dotted lines in (e-g). The myofibers were quantified on the plot in (l). Statistically significant differences were determined by unpaired two-tailed Student's t-test (* p = 0.016, ** p = 0.005, n=5 biologically independent embryos per condition).

(m-p) Zebrafish embryos were either exposed to DMSO vehicle control (m), (-)-Blebbistatin (n), or subjected to CRISPR/Cas9-mediated editing of *ttn* (o) stained for MyHC and Hoescht to analyze myofiber organization and nuclear amounts in the AM muscle at 81 hpf. The MyHC-positive region was segmented to reveal muscle cell nuclei in 3D (m', n', o'). Nuclei count quantifications are shown in (p). Statistically significant differences were determined by unpaired two-tailed Student's t-test (*, p = 0.0487, n= 4 control embryos and 5 *ttn* crispant embryos).

(q-t) A cross-sectional image through the am muscle of control embryos (q), blebbistatin-treated (r), and titin crispant (two examples are shown in s and s'). The myofiber were quantified on the plot in (t). Statistically significant differences were determined by unpaired two-tailed Student's t-test (*, p = 0.002, n=4 embryos per condition).

Scale bars; (a-d), 30 µm. (a'-d'), 10 µm. (e), (i), (m) and (q), 20 µm.

Effects of Wnt/PCP inhibition on developing zebrafish muscles and cartilages

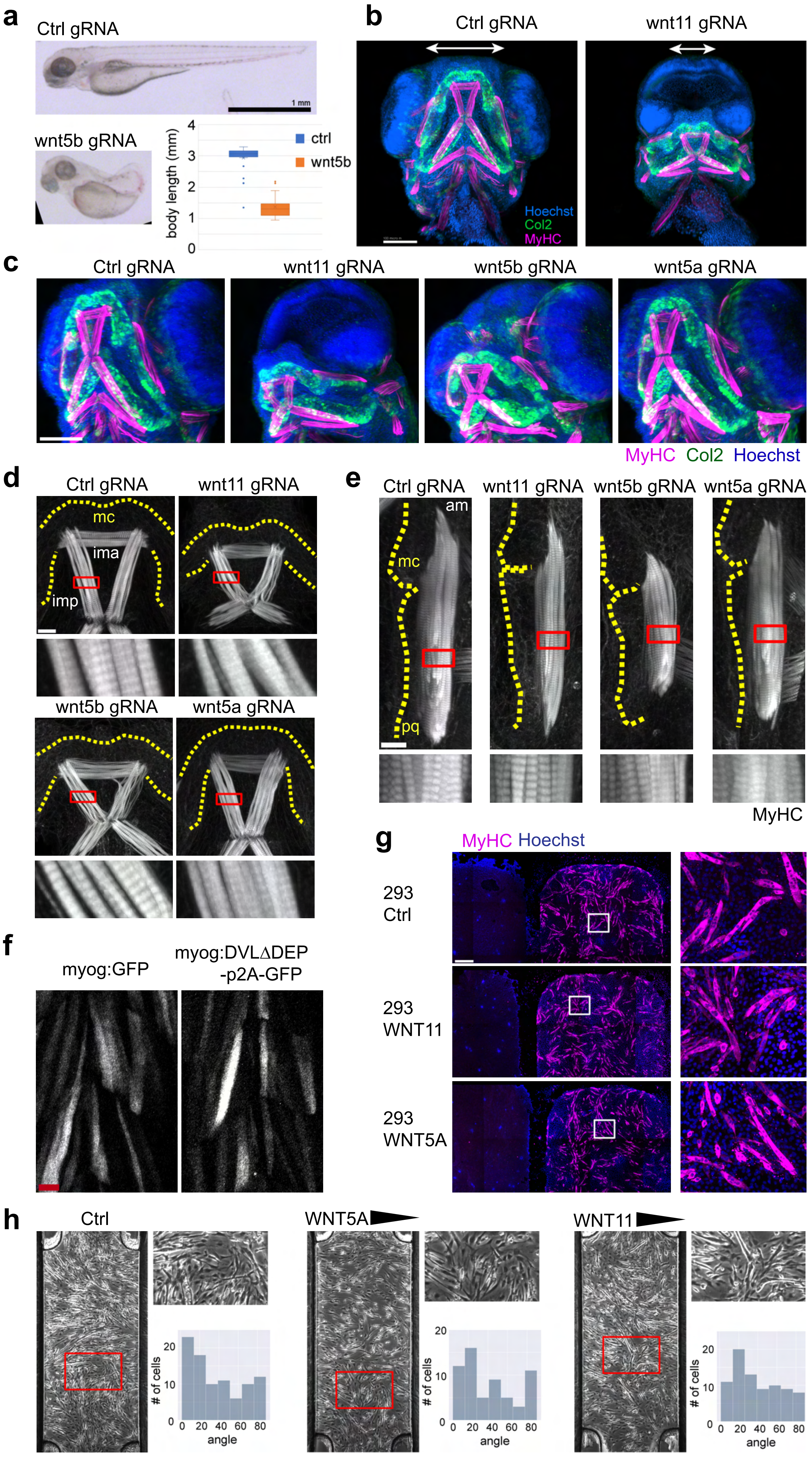

Supplementary Figure 7

Supplementary Figure 7. Wnt11 signaling is not required for effective muscle polarization in zebrafish embryos.

(a-e) Embryos were injected with control-gRNA or gRNA targeting the indicated *WNT* gene, and visualized for the body length (a, quantification in lower right), distance between eyes (b), facial muscle and cartilage (c), or individual myofibrils (d-e). Cartilage (mc and pq) is outlined with dashed yellow lines.

(f) Embryos were injected with GFP or dysfunctional mutant of human Disheveled 3 followed by p2A-GFP, both of which were placed after the promoter of muscle-specific gene, *myogenin*. Muscle cells expressing the gene cassettes in trunk region are visualized with GFP.

(g) 293 cells were seeded and transfected with pCAG-GFP, pCMV6-mWNT5a (MR205939, ORIGENE), or pcDNA3.1-hWNT11 (16758, Addgene). Next day transfected cells were re-seeded in the left side of 2 well cell culture-insert (80209, ibidi). C2C12 cells were seeded on the right side of the insert. The bottom of both sides were coated with type I collagen (Corning). After 24 hours of cell seeding, the insert was removed and differentiation was induced. Cells after 3 days of differentiation are visualized with anti-MyHC and Hoechst staining.

(h) C2C12 cells were plated on an Ibidi microfluidic chip (#80326) and exposed to a gradient of the indicated WNT protein. Cell orientation was quantified and shown in the histogram.

Scale bar; (a), 1mm. (b-c), 100 μm. (d-f), 20 μm. (g), 500 μm.

Inhibition of stretch-induced ion channel in stretched C2C12 cells

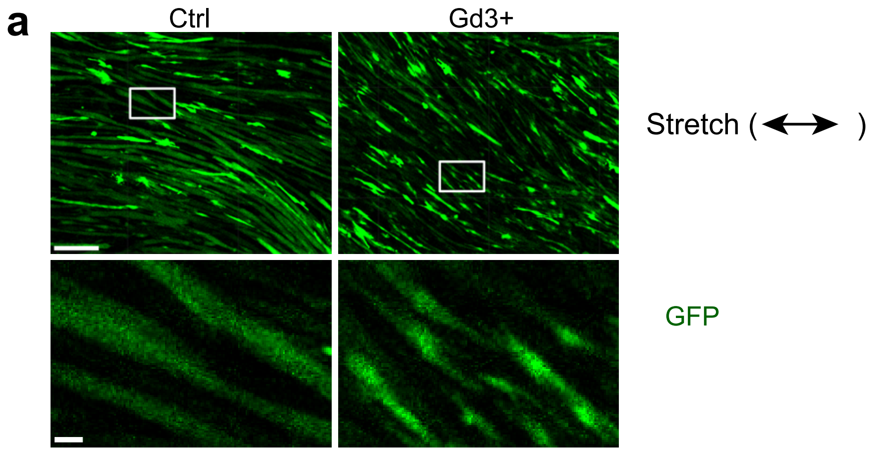

Inhibition of stretch-induced ion channel in zebrafish

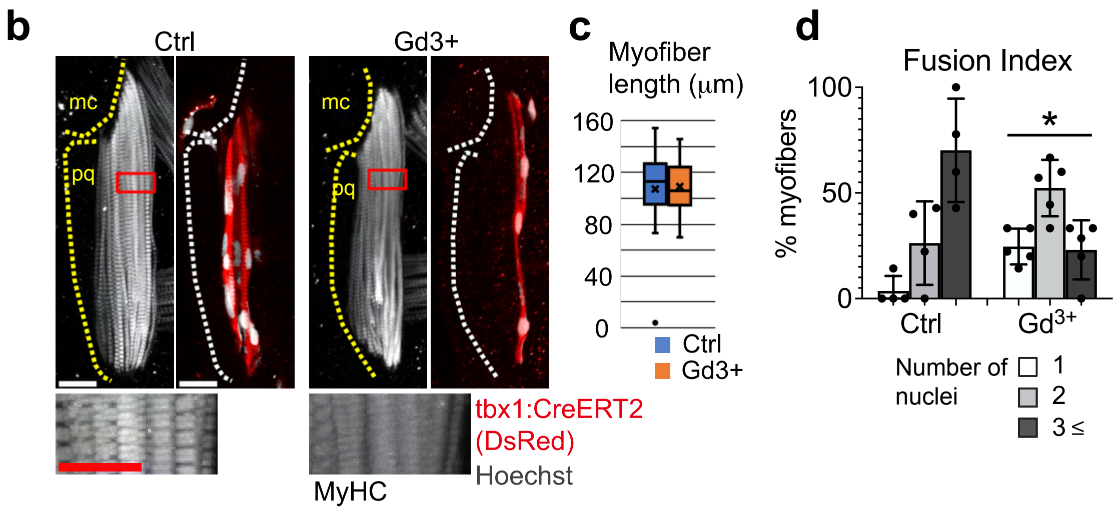

Supplementary Figure 8

Supplementary Figure 8. Blockade of mechanosensory ion channels does not cause pronounced effects on muscle polarization.

(a) C2C12 cells were seeded on laminin-coated PDMS membrane, and transfected with GFP. Cells were treated with Gd^{3+} (25 μM) from 16 hours of differentiation, and then stretched 20% from 20 hours of differentiation. Cells are visualized with GFP at 3.5 days of differentiation. Mock-treated cells are served as control.

(b) Zebrafish embryos were treated with Gd^{3+} (200 μM) from 48 hpf. Myofibrils (MyHC) and individual muscle cells (DsRed) with internal nuclei are visualized at 81 hpf. The region of red box is magnified. Cartilage (pq and am) is outlined with dashed yellow lines.

(c) The length of individual muscle cells is quantified and shown in box plot (n=35 and 29 for control and $Gd3^{+}$-treated, respectively). Box plot = median bounded by IQR, whiskers = 1.5x IQR.

(d) Muscle cells were classified according to the number of nuclei and the percentage of muscle cells is shown. Values are means of n=4 biologically independent control embryos and 5 $Gd3^{+}$-treated embryos ±SD. *, P=0.0009, two-sided Fisher's exact test.

Abbreviations; Gd^{3+}, gadolinium. PDMS, polydimethylsiloxane. GFP, green fluorescent protein. hpf, hours post fertilization. MyHC, Myosin heavy chain. pq, palatoquadrate. am, adductor mandibulae. IQR, interquartile range. SD, standard deviation.

Scale bar; (a) 200 μm. Magnified part of (a), 20 μm. (b), 20 μm. Magnified part of (b), 5 μm.

Expression of b1 integrins in the developing craniofacial region

Effect of b1 integrin loss-of-function on myocyte polarity and fusion

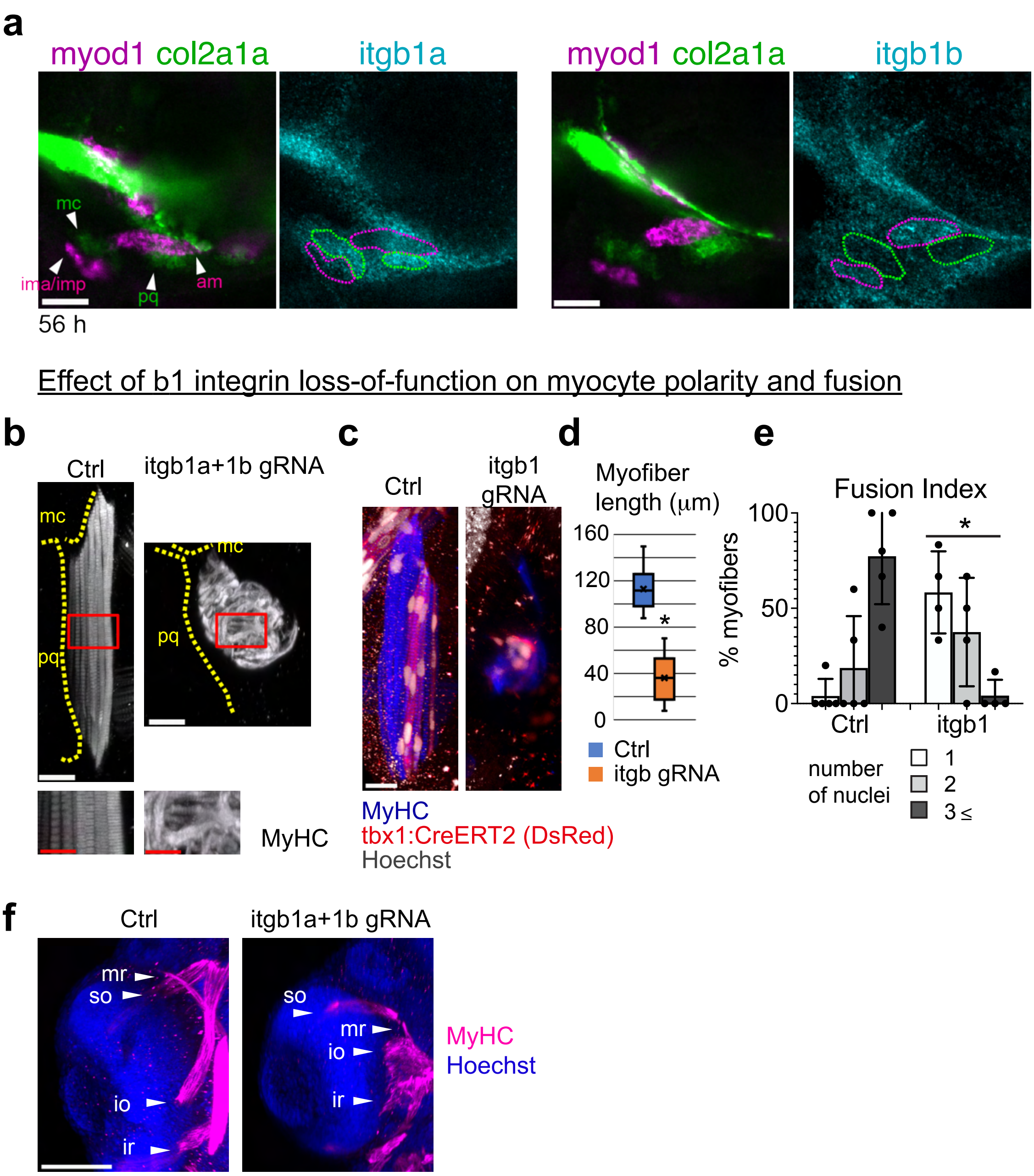

Supplementary Figure 9

Supplementary Figure 9. Integrin adhesion is important for myocyte polarization in zebrafish embryos.

(a) Analysis of gene expression of *itgb1a* and *itgb1b*, in conjunction with *myod1* and *col2a1a,* assessed by HCR in 56 hpf zebrafish embryos. Images are representative of 5 embryos.

(b) Embryos were injected with control-gRNA or gRNA targeting *Itgb1a* plus *Itgb1b*. Myofibrils (MyHC) are visualized at 81 hpf. The region of red box is magnified. Cartilage (mc and pq) is outlined with dashed yellow lines.

(c) Embryos were injected with control-gRNA or gRNA targeting *Itgb1a* plus *Itgb1b*. Individual muscle cells (DsRed) with internal nuclei are visualized at 81 hpf.

(d) The length of individual muscle cells is quantified and shown in box plot. *, $P<0.0001$, unpaired two-tailed Student's t test (n=27 and 17 control and crispant embryos, respectively). Box plot shows median bounded by IQR, whiskers = 1.5x IQR.

(e) Muscle cells were classified according to the number of nuclei and the percentage of muscle cells is shown. Values are means of 5 control and 4 crispant embryos ±SD. *, $P<0.0001$, two-sided Fisher's exact test.

(f) Embryos were injected with control-gRNA or gRNA targeting *Itgb1a* plus *Itgb1b*. The structure of eye muscle.

Abbreviation; mr, medial rectus. so, superior oblique. io, inferior oblique. ir, inferior rectus. IQR, interquartile range.

Scale bar; (a), 50 μm. (b) and (c), upper, 20 μm. (b), lower, 5 μm. (f), 100 μm.

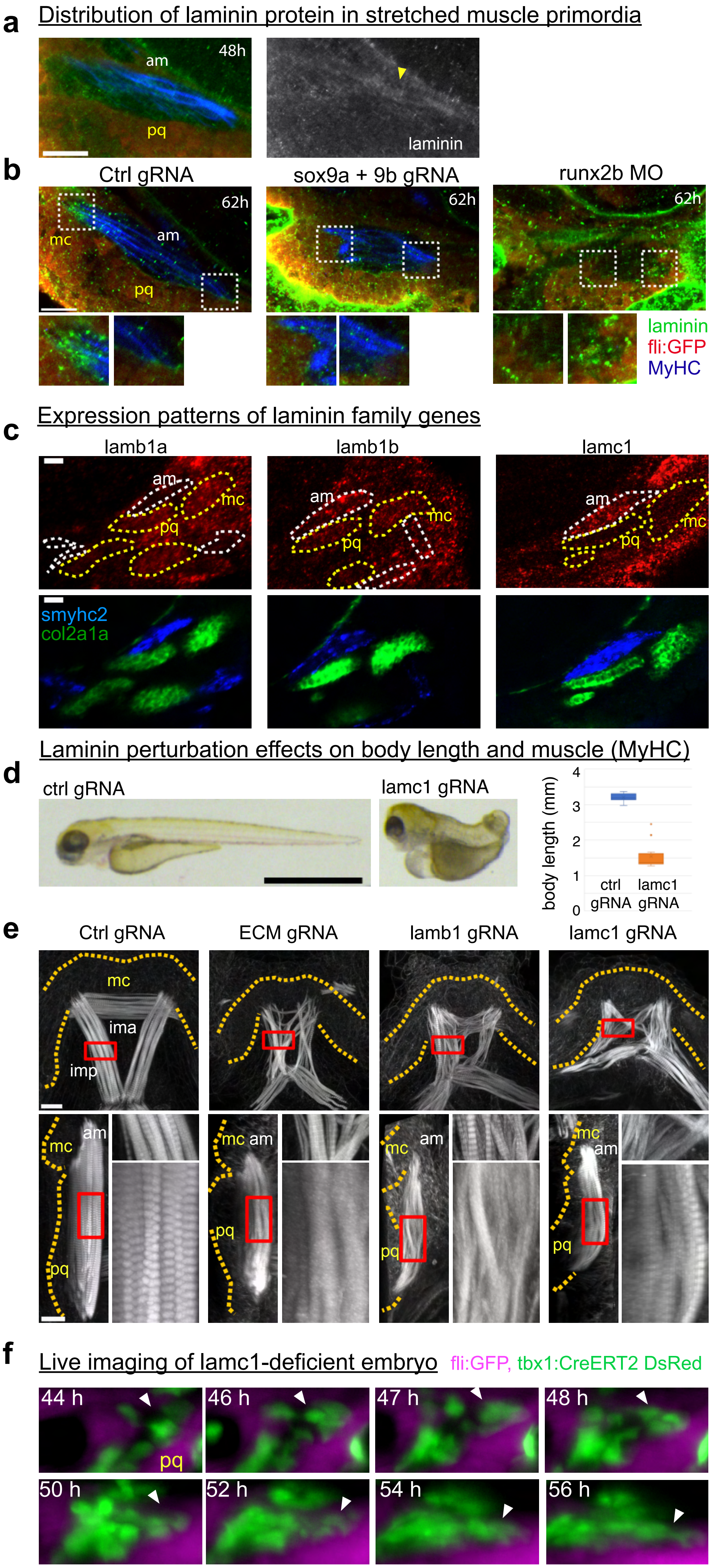

Supplementary Figure 10

Supplementary Figure 10. Extra-cellular matrix components provide myocyte attachment points to guide cell polarization.

(a) Non-treated embryo (48 hpf) were examined for the localization of laminin protein. Images are 1 μm oblique slices. The yellow arrowhead at 48 hpf indicates the accumulation of laminin protein in the muscle area.

(b) control and cartilage-less embryos (62 hpf) were examined for the localization of laminin protein. Images are 1 μm oblique slices. The box area is magnified and shown below.

(c) mRNA of the indicated laminin genes (red) are shown with those of *Smyhc2* and *Col2a1a* at 56 hpf. The area of muscle and cartilage is outlined with white and yellow dashed lines, respectively. Images are 25 μm oblique slices.

(d) Brightfield images of embryos injected with control-gRNA or gRNA targeting *Lamc1*. Body length quantification is shown on the right.

(e) The myofibrils of embryos injected with control-gRNA or gRNA targeting the indicated genes are shown. Boxed area is magnified. Cartilage (pq and am) is outlined with dashed yellow lines. lamb1 gRNA; gRNA targeting *Lamb1a* plus *Lamb1b*. ECM gRNA; gRNA targeting *Col2a1a*, *Col2a1b*, *Acana*, *Acanb*, plus *Comp*.

(f) Differentiating muscle and cartilaginous cells were tracked in *Lamc1* gRNA-injected embryos. Images are shown with 18 μm oblique slices. Arrowheads indicate differentiating muscle cells. See also Supplementary Video 16.

Scale bar; 20 μm except for (d), 1mm.

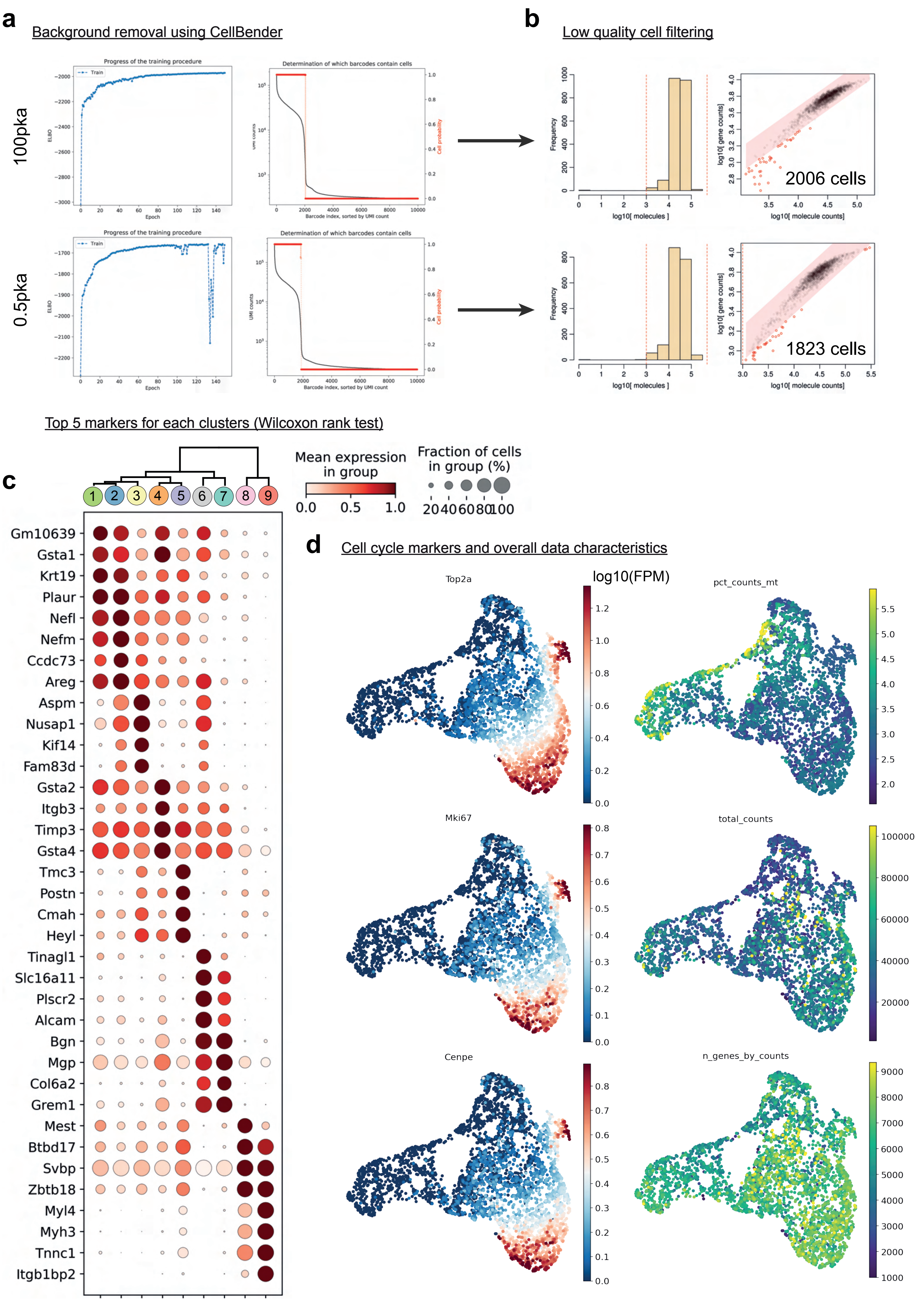

Supplementary Figure 11

Supplementary Figure 11. Quality controls for the single-cell transcriptomics dataset.

(a) For both conditions, resulting plots from the run of the RNA background removal performed on the unfiltered raw count matrices, with ELBO plots showing convergence of the algorithm (left), and barcode rank plot with assigned probability of a droplet to be a cell or not (right).

(b) Cell filtering strategy. c. Dot plot showing the top 5 markers for each clusters, markers were ordered by p-values from Wilcoxon rank test, and the following filtering were applied: being expressed at its highest and by more than 80% of the cells in the given cluster, min log2fold change of 1. For each variable, values are standardized between 0 and 1. d. UMAP plots of main cell cycle markers (left column). UMAP plots of general characteristics of the dataset (right column) with: proportion of mitochondrial gene expressed (top). Total number of transcript (middle). Number of detected genes (bottom).

(c) Top 5 marker genes of each cluster. Hue of each circle shows expression intensity per cell, on average within a cluster. Size of the circle represents the fraction of cells in a cluster highly expressing the marker gene.

(d) Left, cell cycle markers that are representative of the overall cell cycle score from Figure 8j. Right, overall data characteristics are shown.

Additional population marker genes

a

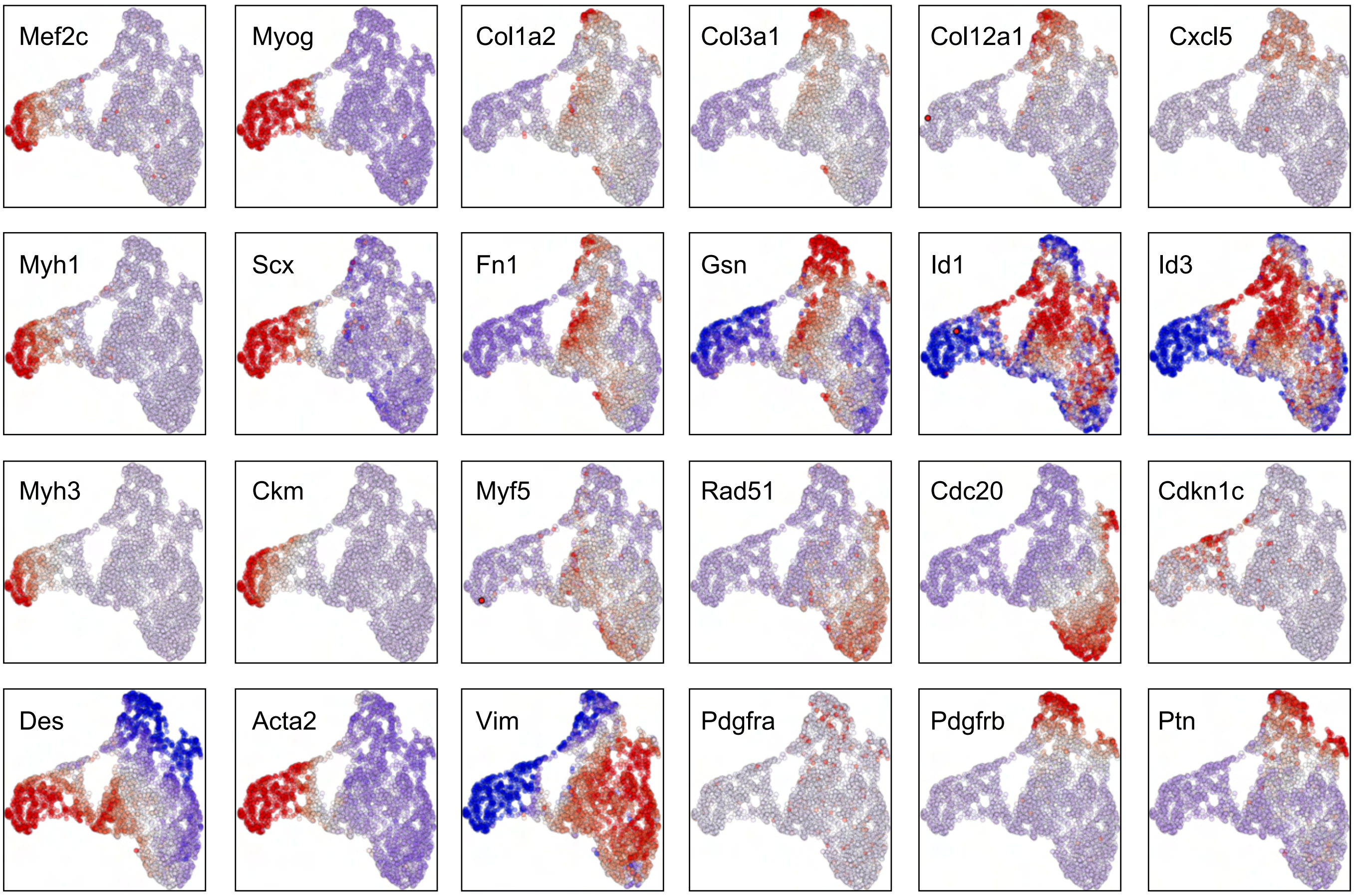

Phase portraits of high velocity genes in bridge 1

b

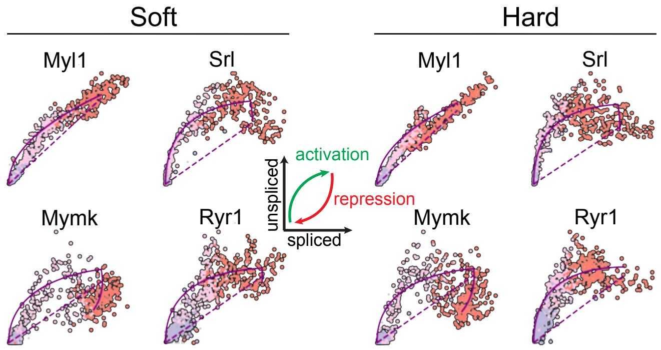

GO terms, first PC score

c

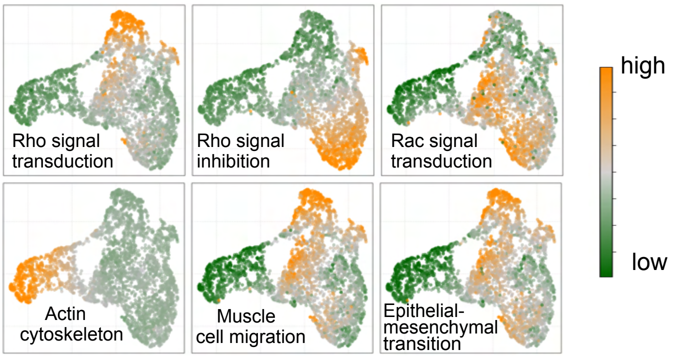

Supplementary Figure 12

Supplementary Figure 12. Cell type annotation of the single-cell clusters.

(a) Additional markers that were used to determine the identity of clusters.

(b) Scatterplots showing the levels of spliced versus un-spliced transcripts of selected genes that are significantly associated with the RNA velocity of bridge-like cell clusters.

(c) GO terms found to be significantly differentially regulated in cluster 4, a cluster with above-average expression of adhesion and mechanosensitive genes, and is over-represented by cells from the soft fraction. Orange indicates increased pathway activation, green indicates less pathway activation.

Top 18 high velocity genes from bridge 1

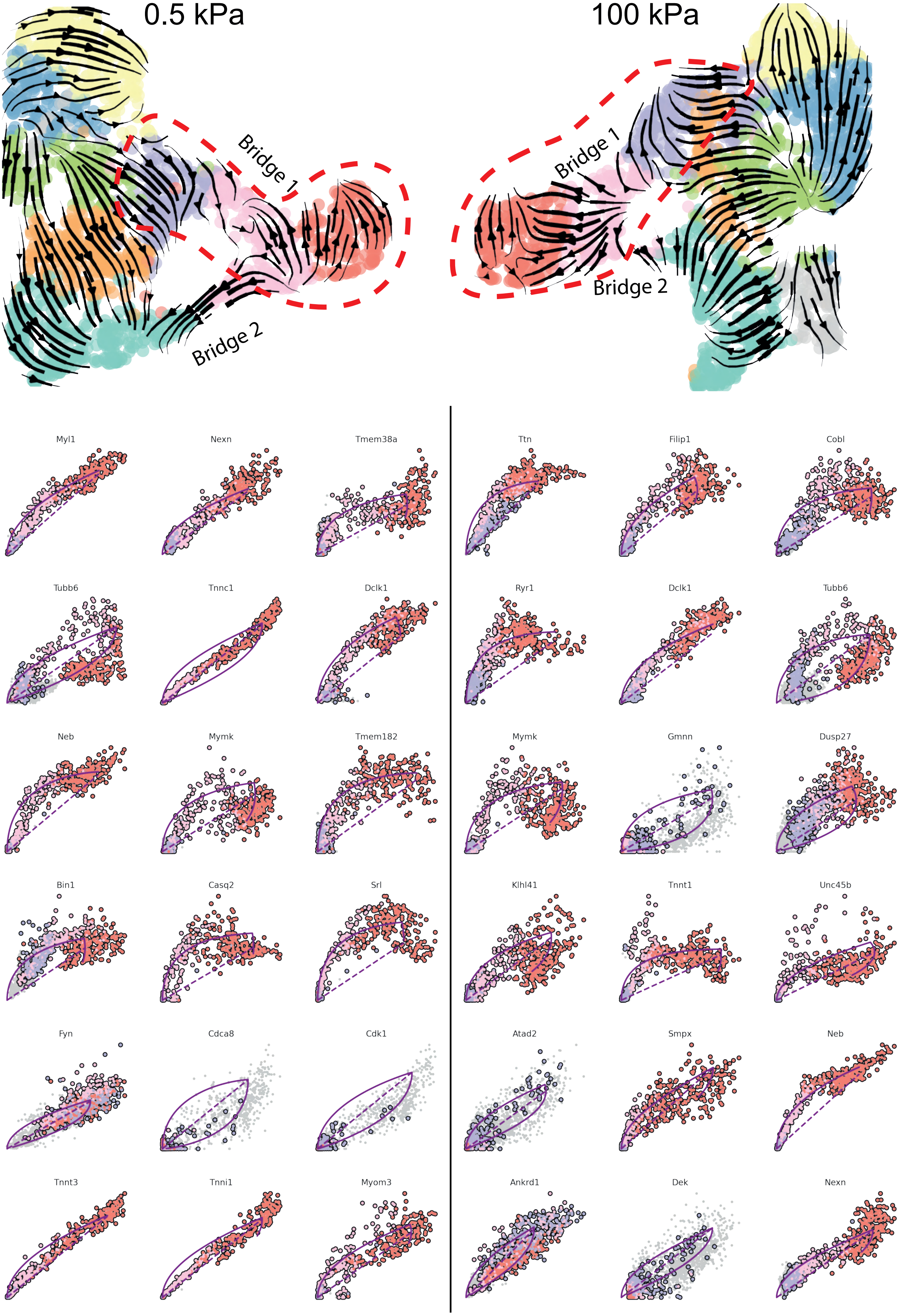

Supplementary Figure 13

Supplementary Figure 13. Phase portraits of high velocity genes in the trajectory between desmin-expressing myoblasts and mature myocytes (Bridge 1).

RNA velocity-based phase portraits for Bridge 1 myocyte differentiation *in vitro*. (left) UMAP embeddings of both conditions with mapped RNA velocity represented as a streamplot. (right) Phase portrait (x-axis: spliced, y-axis: unspliced) of the top 18 high velocity genes defining the bridge 1. Selection was performed by performing two-sided Welch t-test with overestimated variance on velocity expression of genes having a minimum likelihood of 0.4. T-test was performed on the combination of clusters 8 and 5 versus the rest.

Top 18 high velocity genes from bridge 2

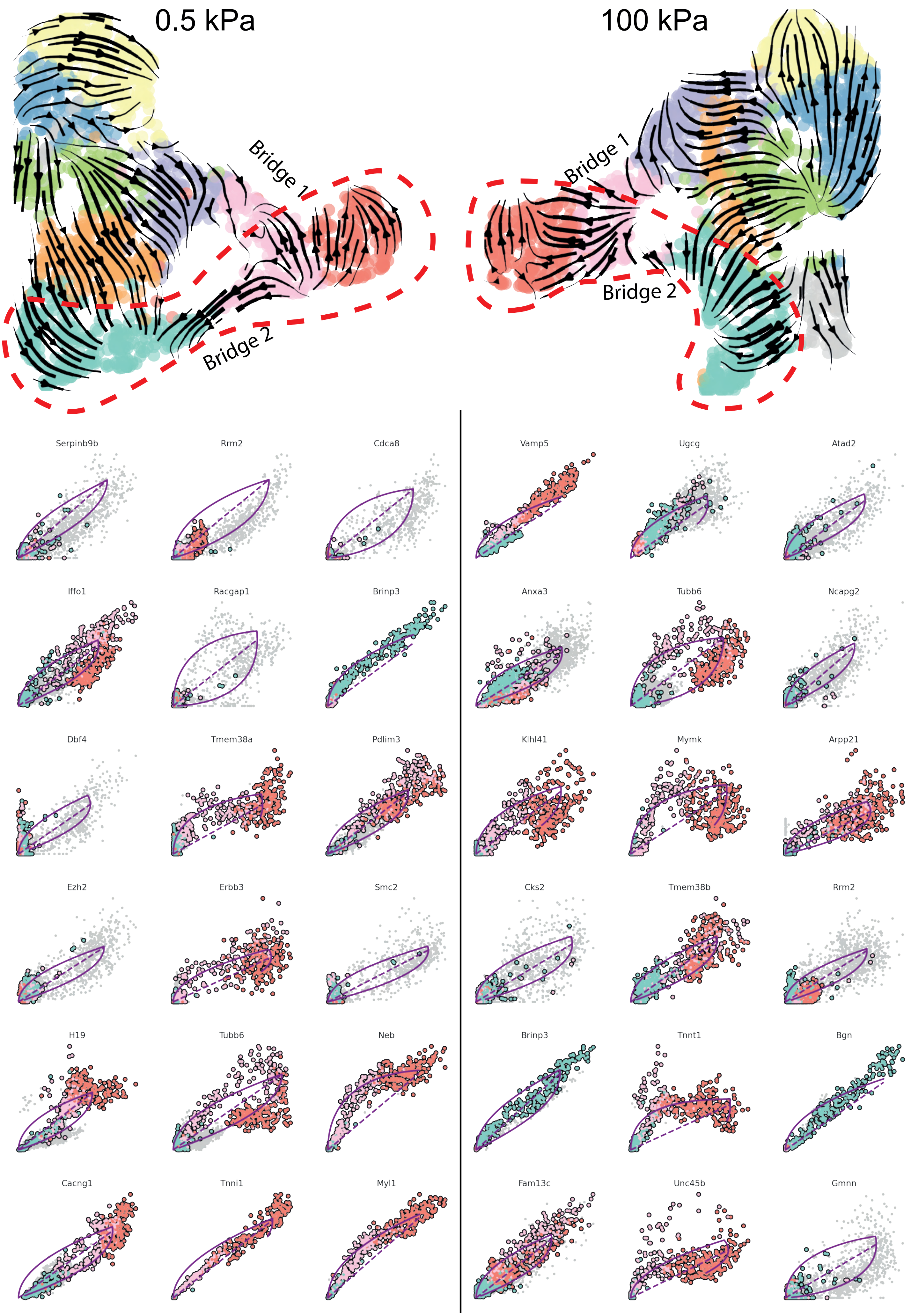

Supplementary Figure 14

Supplementary Figure 14. Phase portraits of high velocity genes in the trajectory between myocytes and myofibroblasts (Bridge 2).

RNA velocity-based phase portraits for Bridge 2 myocyte differentiation *in vitro.* (left) UMAP embeddings of both conditions with mapped RNA velocity represented as a streamplot. (right) Phase portrait (x-axis: spliced, y-axis: unspliced) of the top 18 high velocity genes defining the bridge 2. Selection was performed by performing two-sided Welch t-test with overestimated variance on velocity expression of genes having a minimum likelihood of 0.4. T-test was performed on the combination of clusters 8 and 7 versus the rest.

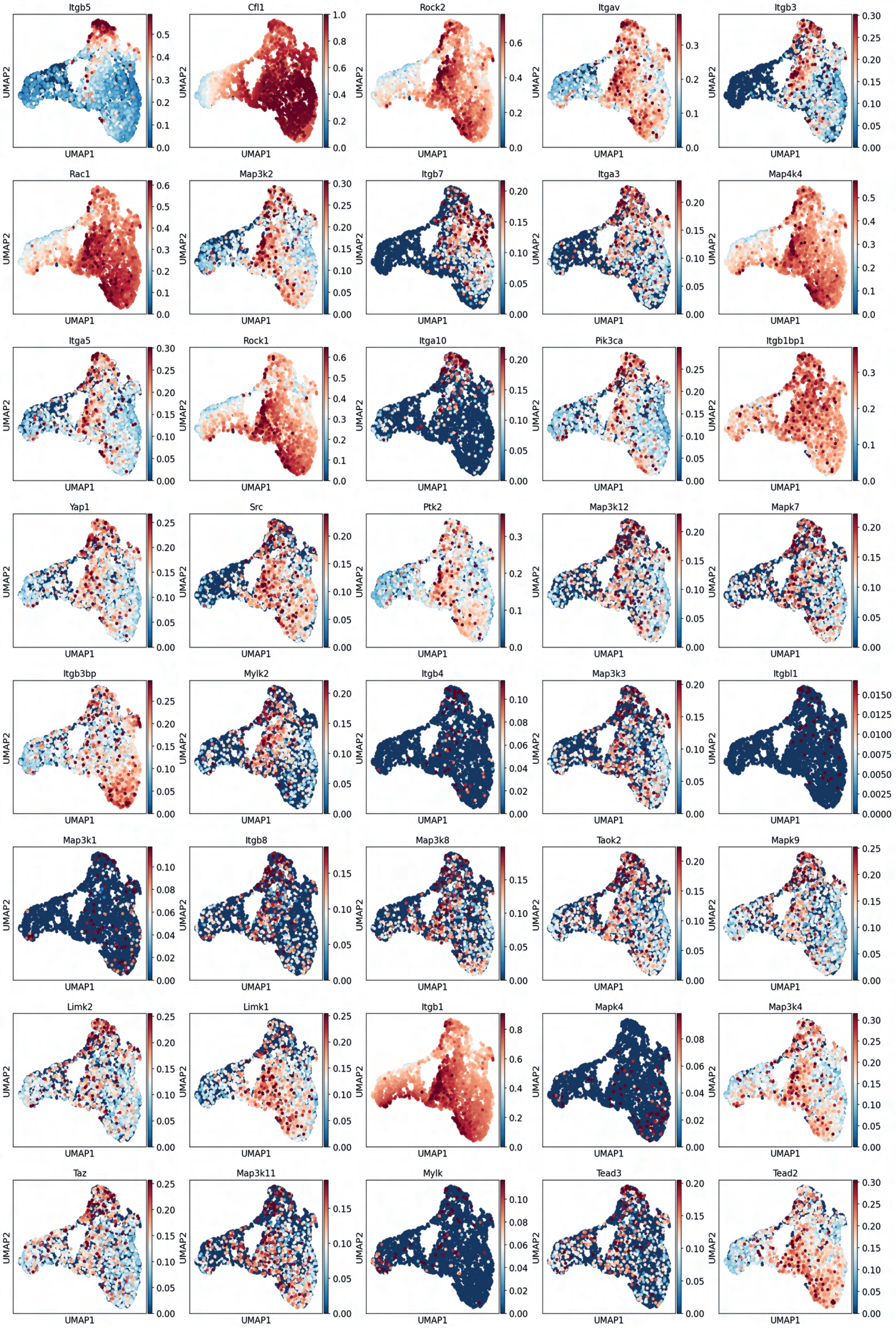
Itgb5
Cfl1
Rock2
Itgav
Itgb3
Rac1
Map3k2
Itgb7
Itga3
Map4k4
Itga5
Rock1
Itga10
Pik3ca
Itgb1bp1
Yap1
Src
Ptk2
Map3k12
Mapk7
Itgb3bp
Mylk2
Itgb4
Map3k3
Itgbl1
Map3k1
Itgb8
Map3k8
Taok2
Mapk9
Limk2
Limk1
Itgb1
Mapk4
Map3k4
Taz
Map3k11
Mylk
Tead3
Tead2
UMAP1
UMAP2

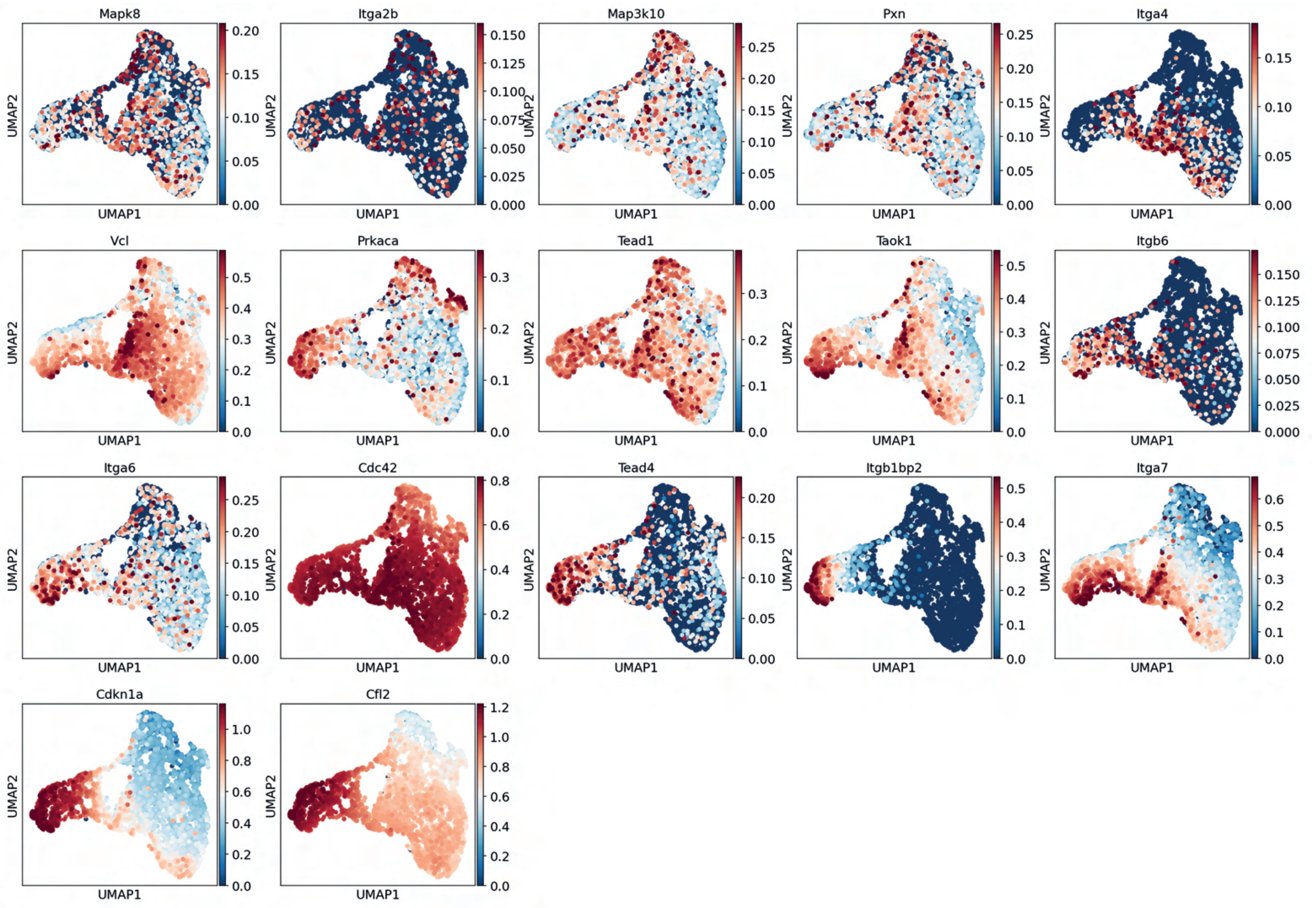
Mapk8
Itga2b
Map3k10
Pxn
Itga4
Vcl
Prkaca
Tead1
Taok1
Itgb6
Itga6
Cdc42
Tead4
Itgb1bp2
Itga7
Cdkn1a
Cfl2
UMAP1
UMAP2

Supplementary Figure 15

UMAP plots of selected markers. Additional UMAP plots from the single cell transcriptomics dataset in Figure 8, showing expression of selected gene markers, with a focus on mechanotransduction-related, extracellular matrix-related, adhesion-related, and JNK signaling pathway-related genes.

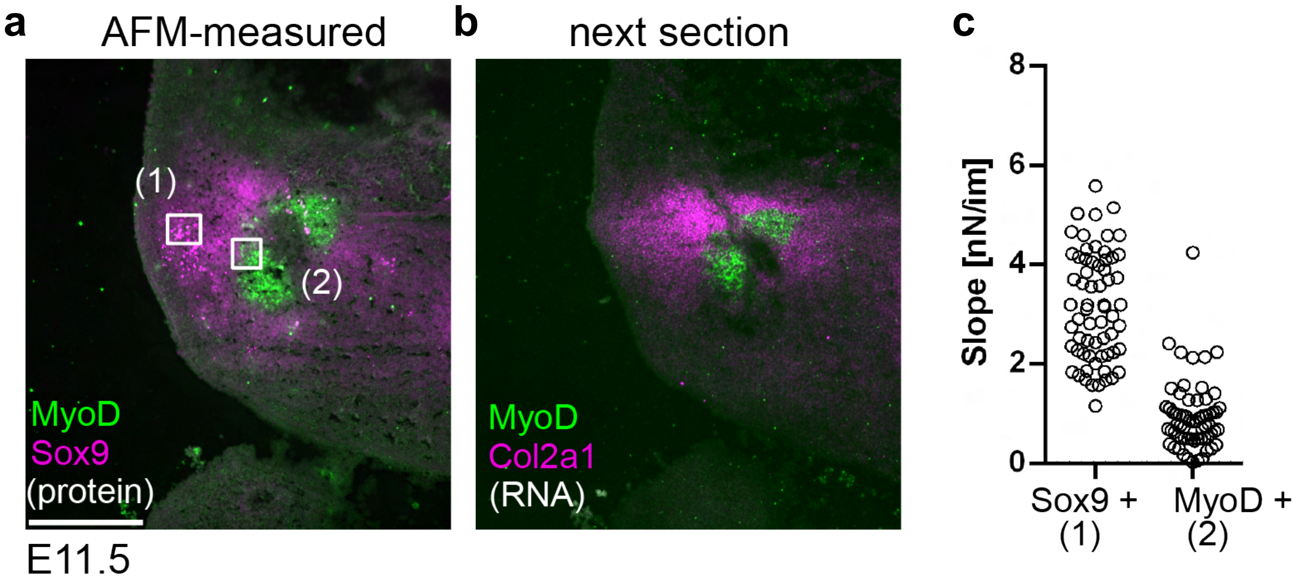

Supplementary Figure 16

Supplementary Figure 16. Atomic force microscopy of tissue sections may reveal the early-emerging differences in mechanical properties of differentiating cartilage and muscle.

Mouse embryonic day 11.5 craniofacial tissue analyzed both by atomic force microscopy (AFM) and fluorescence microscopy for tissue markers. **(a)** The AFM-measured sections also subsequently immunostained for MyoD (muscle) and Sox9 (cartilage). **(b)** The next tissue section was probed by HCR for gene expression. Boxes in (a) represent areas probed with AFM. **(c)** the calculated elastic modulus values for each are shown. Scale bar; 500 μm.

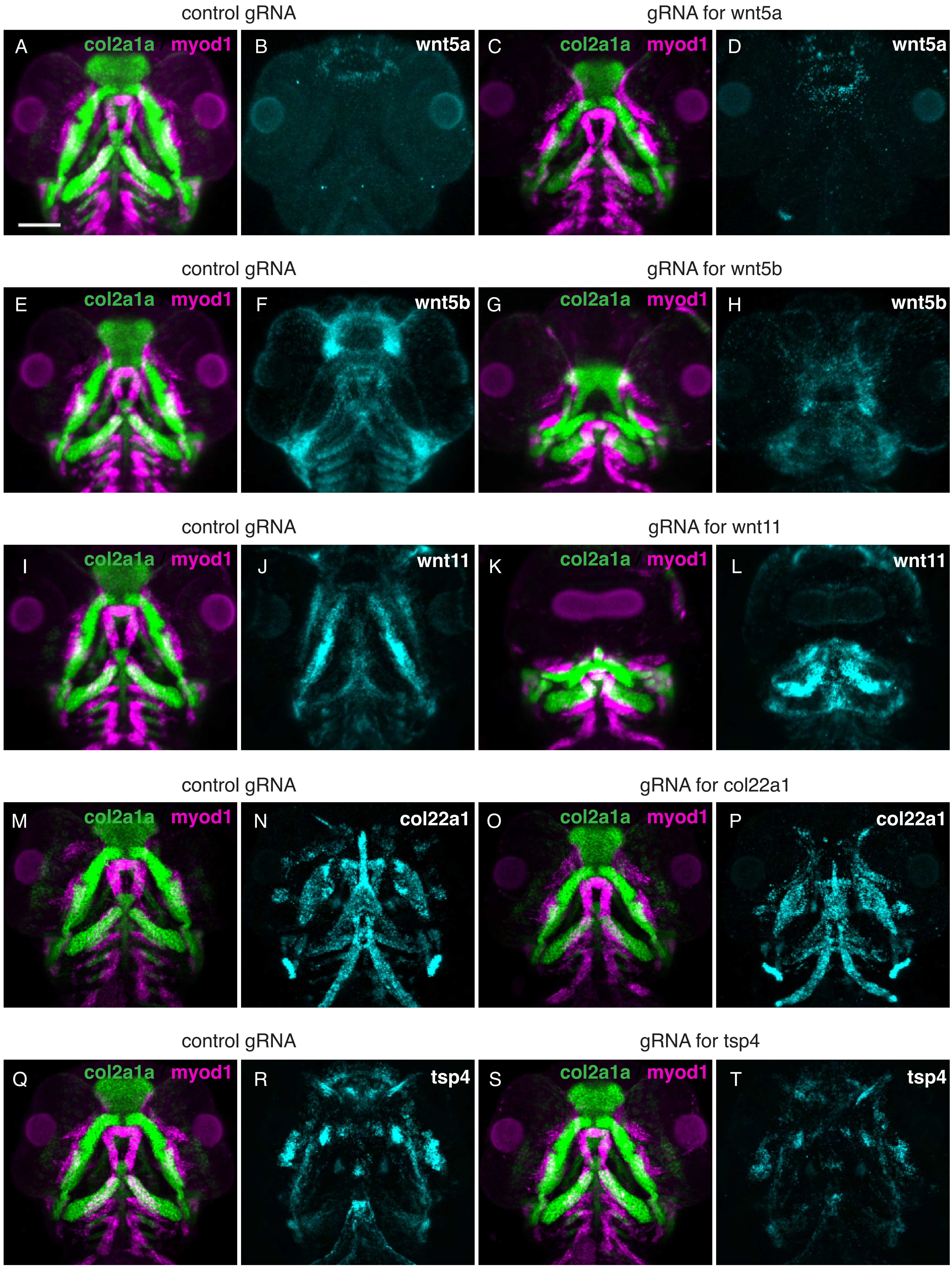

control gRNA
gRNA for wnt5a
A
col2a1a
myod1
B
wnt5a
C
col2a1a
myod1
D
wnt5a
control gRNA
gRNA for wnt5b
E
col2a1a
myod1
F
wnt5b
G
col2a1a
myod1
H
wnt5b
control gRNA
gRNA for wnt11
I
col2a1a
myod1
J
wnt11
K
col2a1a
myod1
L
wnt11
control gRNA
gRNA for col22a1
M
col2a1a
myod1
N
col22a1
O
col2a1a
myod1
P
col22a1
control gRNA
gRNA for tsp4
Q
col2a1a
myod1
R
tsp4
S
col2a1a
myod1
T
tsp4

Supplementary Figure 17.

HCR staining to assess RNA-mediated decay of gene targets in crispant embryos. In situ hybridization of genes targeted by CRISPR/Cas9 technology, with a focus on gene targets that appeared to not have a noticeable effect on myocyte orientation. Target genes include wnt5a (a-d), wnt5b (e-h), wnt11 (i-l), col22a1 (m-p), and tsp4 (q-t). Left panels show embryos injected with control gRNA at 72 hpf, hybridized with HCR probes against cartilage (col2a1a, signal shown in green) and muscle (myod1, signal shown in magenta) versus the target gene (gene name indicated, signal shown in cyan). Right panels show embryos injected with the indicated target gRNA at 72 hpf, hybridized with HCR probes against cartilage (col2a1a, signal shown in green) and muscle (myod1, signal shown in magenta) versus the target gene (gene name indicated, signal shown in cyan). Scale bar, 100 µm.

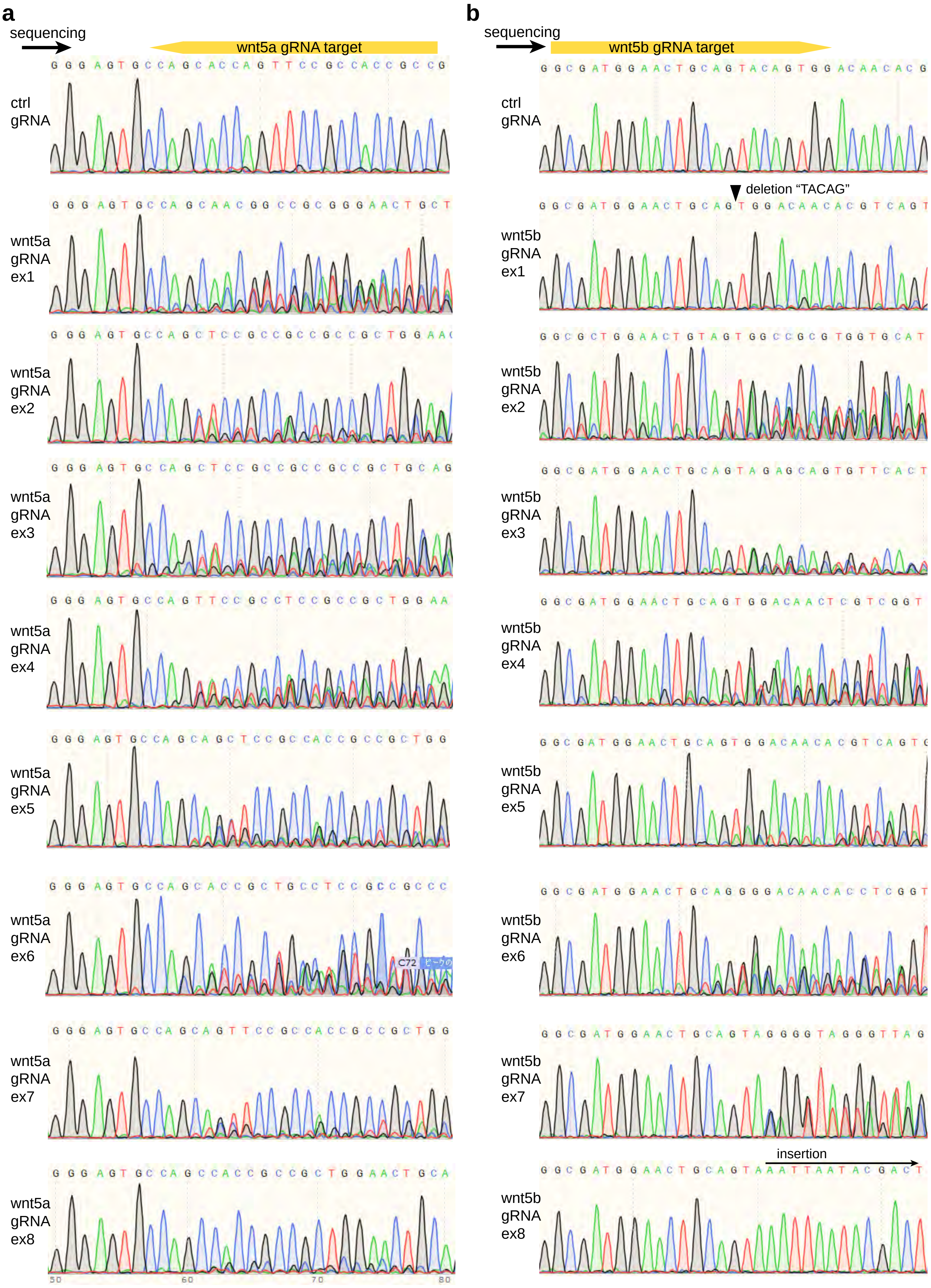
a
sequencing
wnt5a gRNA target
ctrl gRNA
wnt5a gRNA ex1
wnt5a gRNA ex2
wnt5a gRNA ex3
wnt5a gRNA ex4
wnt5a gRNA ex5
wnt5a gRNA ex6
wnt5a gRNA ex7
wnt5a gRNA ex8
50
60
70
80
b
sequencing
wnt5b gRNA target
ctrl gRNA
deletion "TACAG"
wnt5b gRNA ex1
wnt5b gRNA ex2
wnt5b gRNA ex3
wnt5b gRNA ex4
wnt5b gRNA ex5
wnt5b gRNA ex6
wnt5b gRNA ex7
insertion
wnt5b gRNA ex8

c

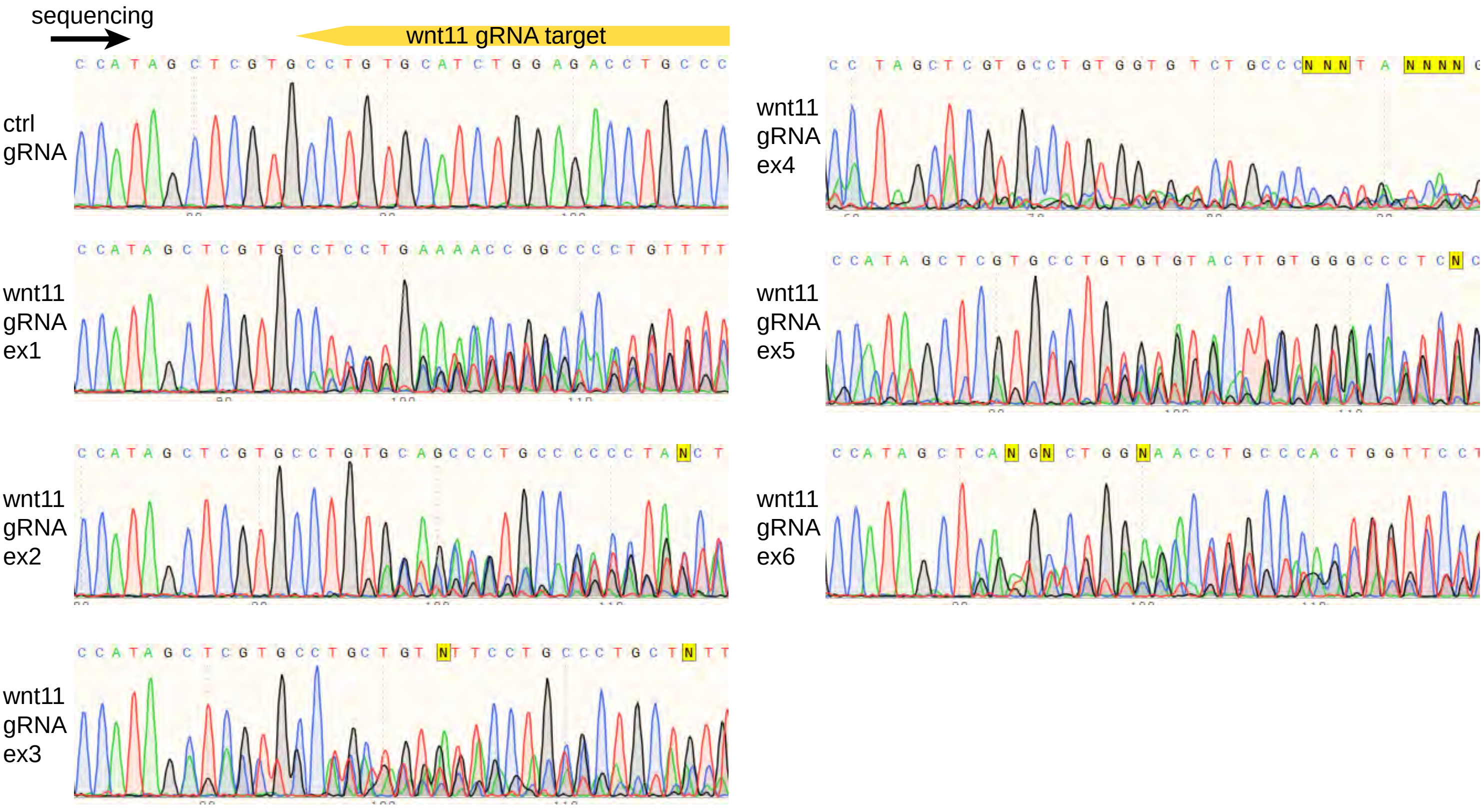
sequencing
wnt11 gRNA target
ctrl gRNA
wnt11 gRNA ex1
wnt11 gRNA ex2
wnt11 gRNA ex3
wnt11 gRNA ex4
wnt11 gRNA ex5
wnt11 gRNA ex6

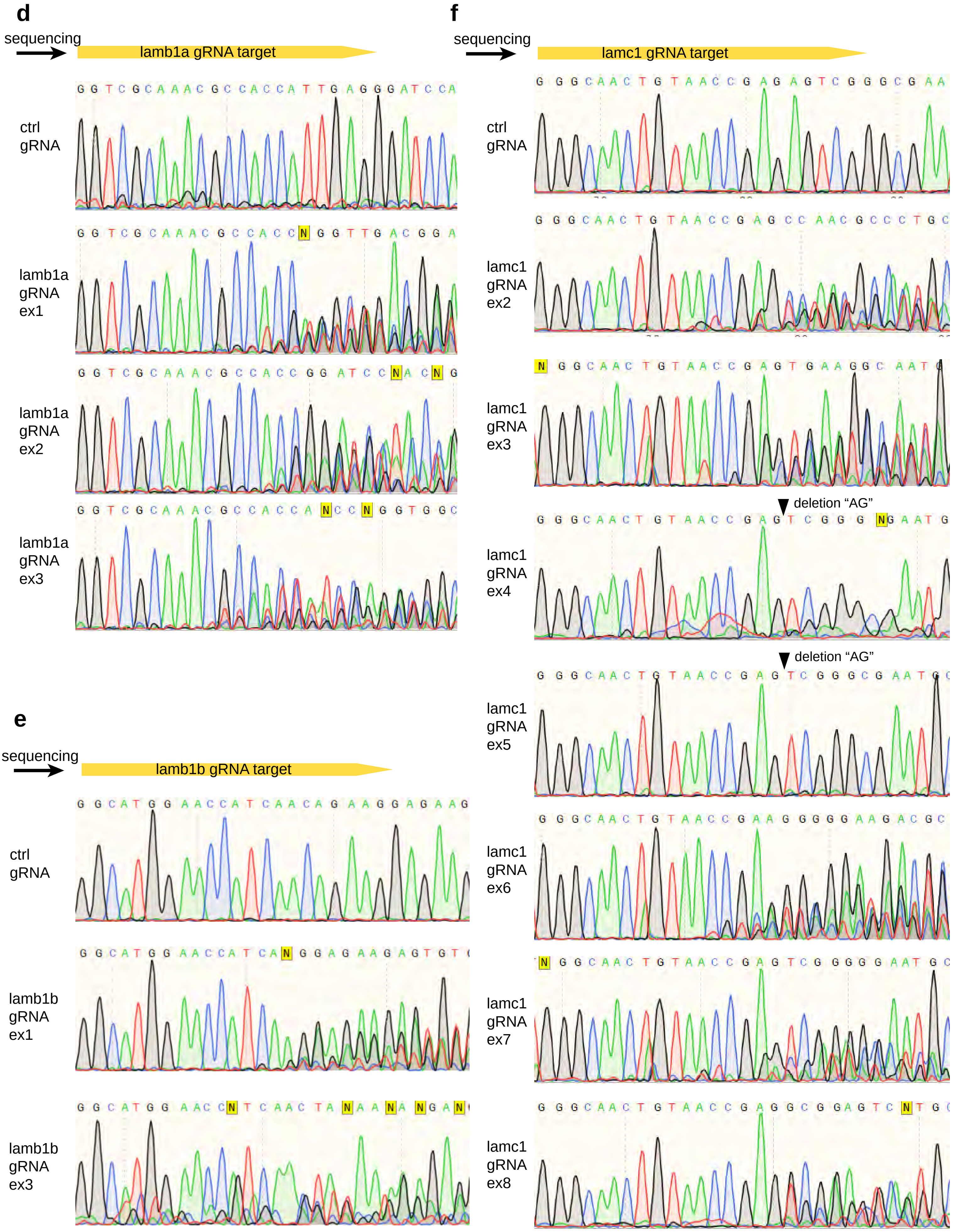
d
sequencing
lamb1a gRNA target
GGTCGCAAACGCCACCATTGAGGGATCCA
ctrl gRNA
GGTCGCAAACGCCACCNGGTTGACGGA
lamb1a gRNA ex1
GGTCGCAAACGCCACCGGATCCNACNG
lamb1a gRNA ex2
GGTCGCAAACGCCACCANCCNGGTGGC
lamb1a gRNA ex3
e
sequencing
lamb1b gRNA target
GGCATGGAACCATCAACAGAAGGAGAAG
ctrl gRNA
GGCATGGAACCATCANGGAGAAGAGTGT
lamb1b gRNA ex1
GGCATGGAACCNTCAACTANAANANGAN
lamb1b gRNA ex3
f
sequencing
lamc1 gRNA target
GGGCAACTGTAACCGAGAGTCGGGCGAA
ctrl gRNA
GGGCAACTGTAACCGAGCCAACGCCCTGC
lamc1 gRNA ex2
NGGCAACTGTAACCGAGTGAAGGCAATG
lamc1 gRNA ex3
deletion “AG”
GGGCAACTGTAACCGAGTCGGGNGAATG
lamc1 gRNA ex4
deletion “AG”
GGGCAACTGTAACCGAGTCGGGCGAATG
lamc1 gRNA ex5
GGGCAACTGTAACCGAAGGGGGAAGACGC
lamc1 gRNA ex6
NGGCAACTGTAACCGAGTCGGGGAATGC
lamc1 gRNA ex7
GGGCAACTGTAACCGAGGCGGAGTCNTG
lamc1 gRNA ex8

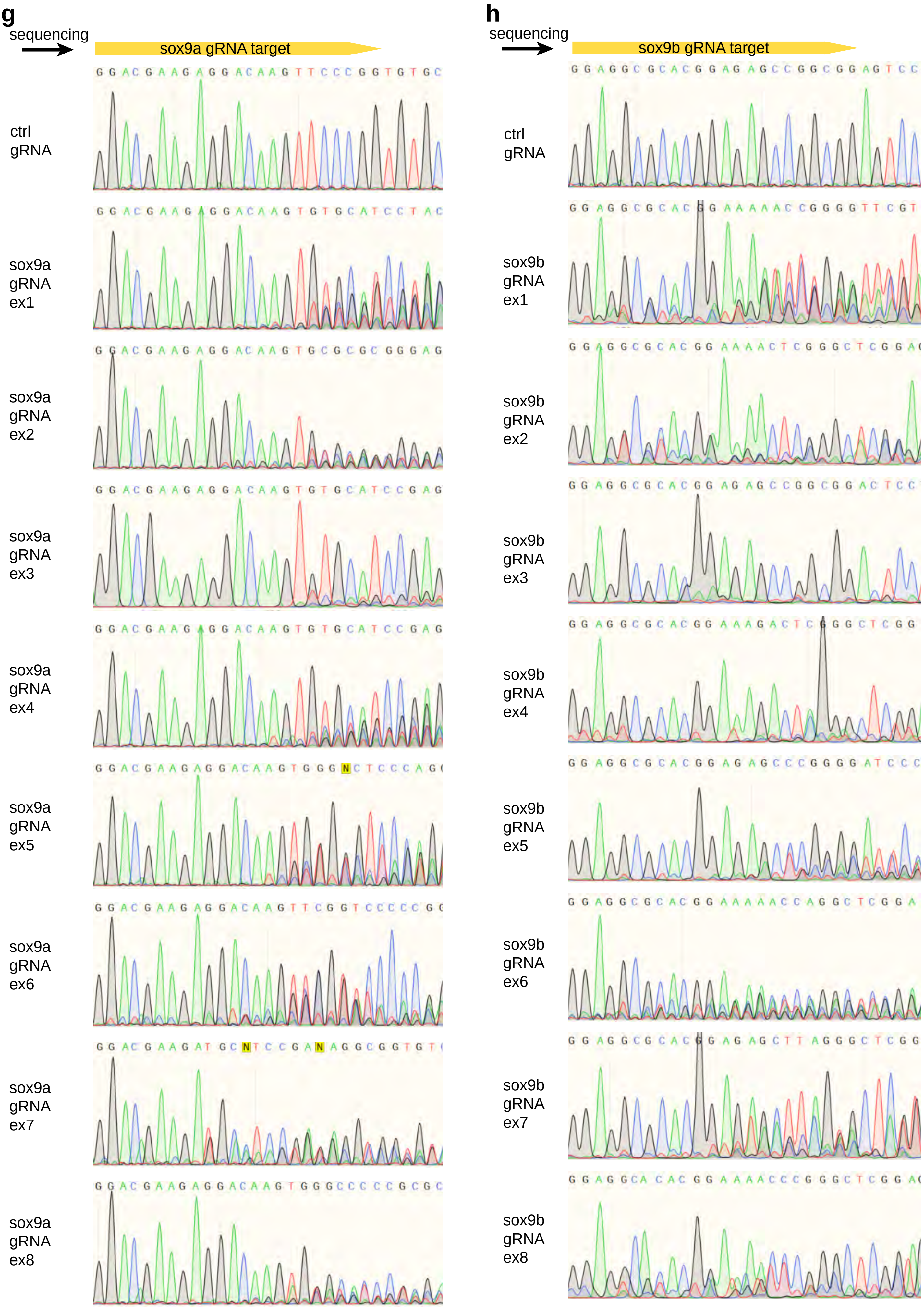
g
sequencing
sox9a gRNA target
ctrl gRNA
sox9a gRNA ex1
sox9a gRNA ex2
sox9a gRNA ex3
sox9a gRNA ex4
sox9a gRNA ex5
sox9a gRNA ex6
sox9a gRNA ex7
sox9a gRNA ex8
h
sequencing
sox9b gRNA target
ctrl gRNA
sox9b gRNA ex1
sox9b gRNA ex2
sox9b gRNA ex3
sox9b gRNA ex4
sox9b gRNA ex5
sox9b gRNA ex6
sox9b gRNA ex7
sox9b gRNA ex8

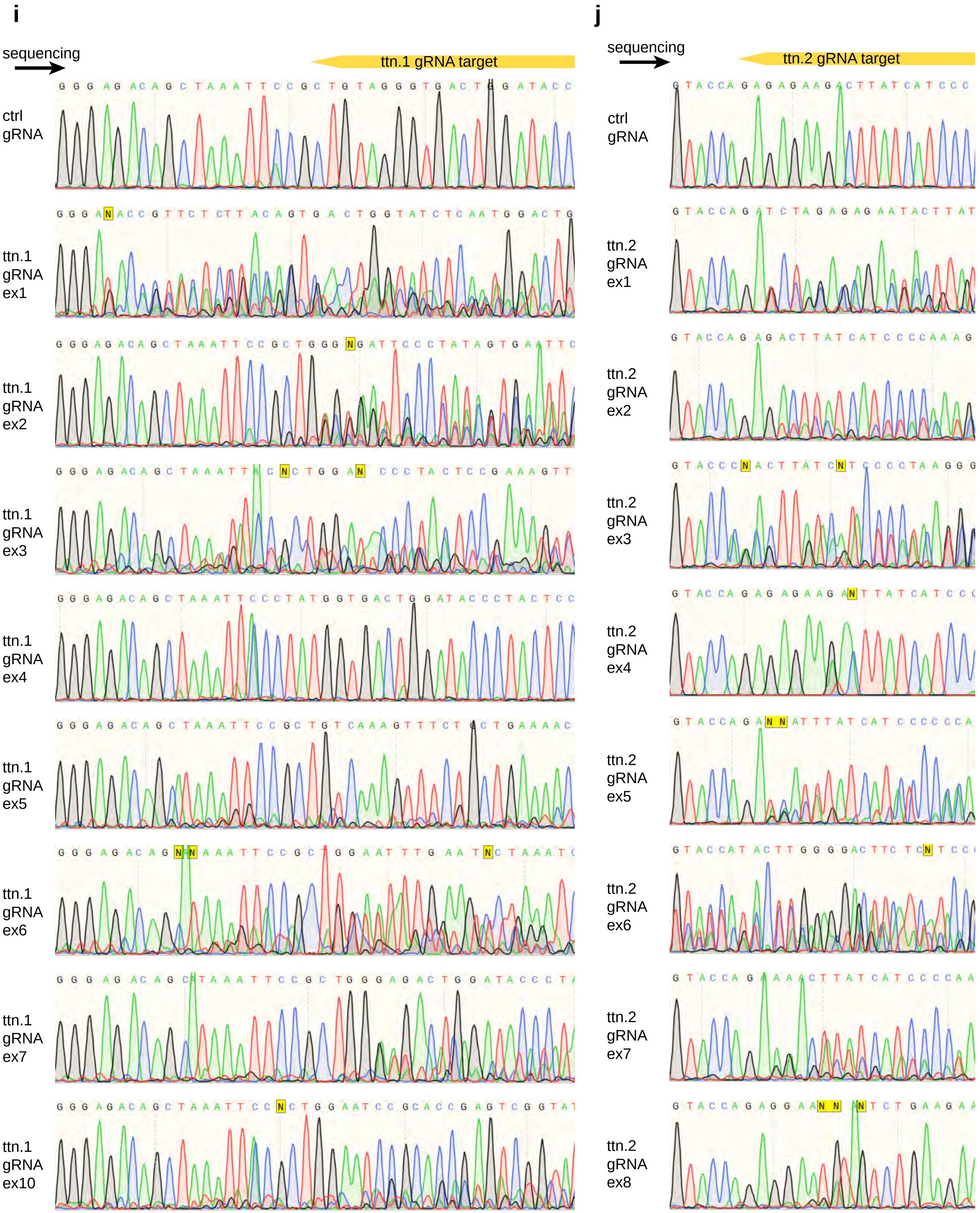
i
sequencing
ttn.1 gRNA target
ctrl gRNA
ttn.1 gRNA ex1
ttn.1 gRNA ex2
ttn.1 gRNA ex3
ttn.1 gRNA ex4
ttn.1 gRNA ex5
ttn.1 gRNA ex6
ttn.1 gRNA ex7
ttn.1 gRNA ex10
j
sequencing
ttn.2 gRNA target
ctrl gRNA
ttn.2 gRNA ex1
ttn.2 gRNA ex2
ttn.2 gRNA ex3
ttn.2 gRNA ex4
ttn.2 gRNA ex5
ttn.2 gRNA ex6
ttn.2 gRNA ex7
ttn.2 gRNA ex8

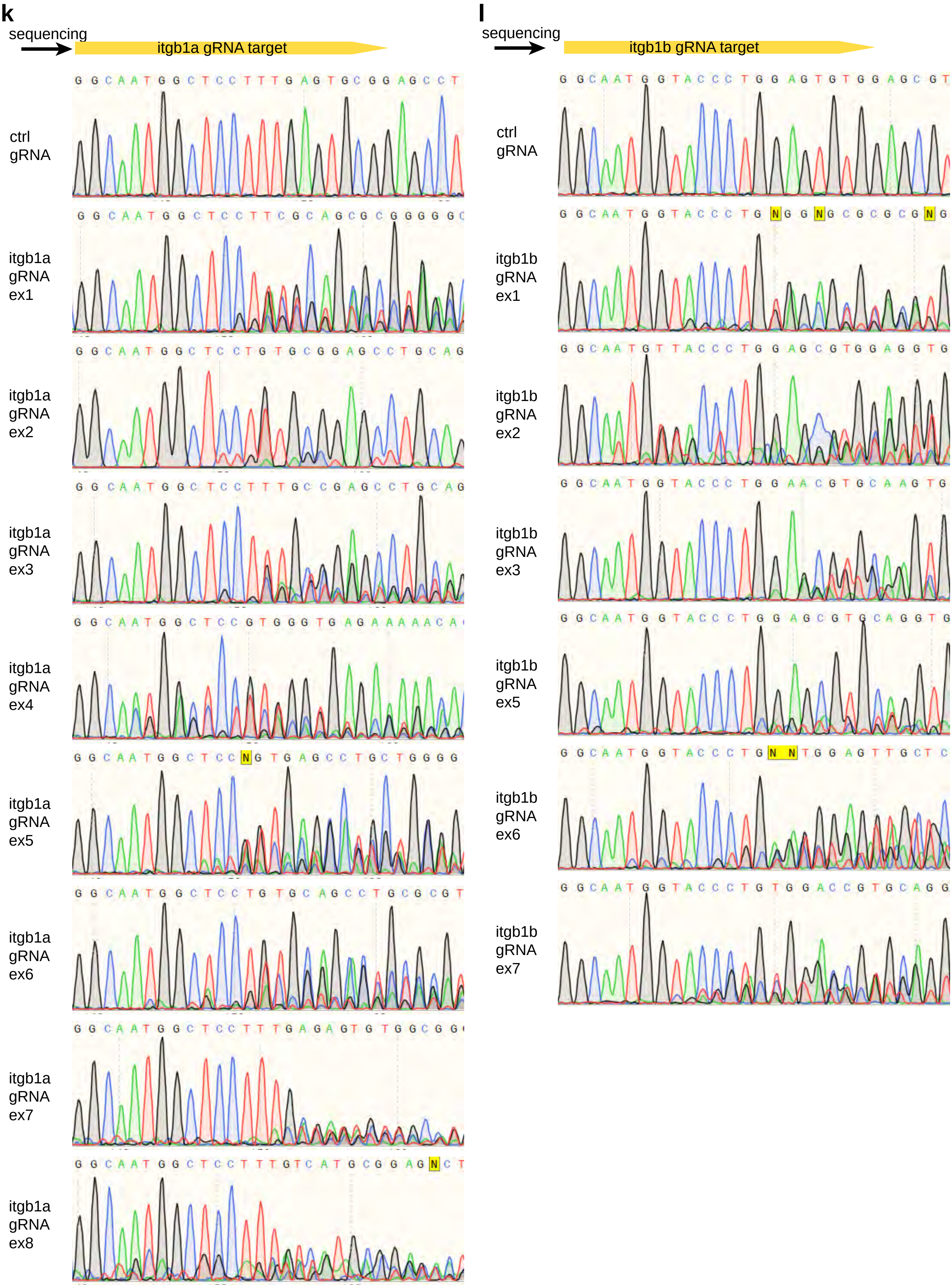
k
sequencing
itgb1a gRNA target
G G C A A T G G C T C C T T T G A G T G C G G A G C C T
ctrl gRNA
G G C A A T G G C T C C T T C G C A G C G C G G G G G C
itgb1a gRNA ex1
G G C A A T G G C T C C T G T G C G G A G C C T G C A G
itgb1a gRNA ex2
G G C A A T G G C T C C T T T G C C G A G C C T G C A G
itgb1a gRNA ex3
G G C A A T G G C T C C G T G G G T G A G A A A A A C A
itgb1a gRNA ex4
G G C A A T G G C T C C N G T G A G C C T G C T G G G G
itgb1a gRNA ex5
G G C A A T G G C T C C T G T G C A G C C T G C G C G T
itgb1a gRNA ex6
G G C A A T G G C T C C T T T G A G A G T G T G G C G G
itgb1a gRNA ex7
G G C A A T G G C T C C T T T G T C A T G C G G A G N C T
itgb1a gRNA ex8
l
sequencing
itgb1b gRNA target
G G C A A T G G T A C C C T G G A G T G T G G A G C G T
ctrl gRNA
G G C A A T G G T A C C C T G N G G N G C G C G C G N G
itgb1b gRNA ex1
G G C A A T G T T A C C C T G G A G C G T G G A G G T G
itgb1b gRNA ex2
G G C A A T G G T A C C C T G G A A C G T G C A A G T G
itgb1b gRNA ex3
G G C A A T G G T A C C C T G G A G C G T G C A G G T G
itgb1b gRNA ex5
G G C A A T G G T A C C C T G N N T G G A G T T G C T C
itgb1b gRNA ex6
G G C A A T G G T A C C C T G T G G A C C G T G C A G G
itgb1b gRNA ex7

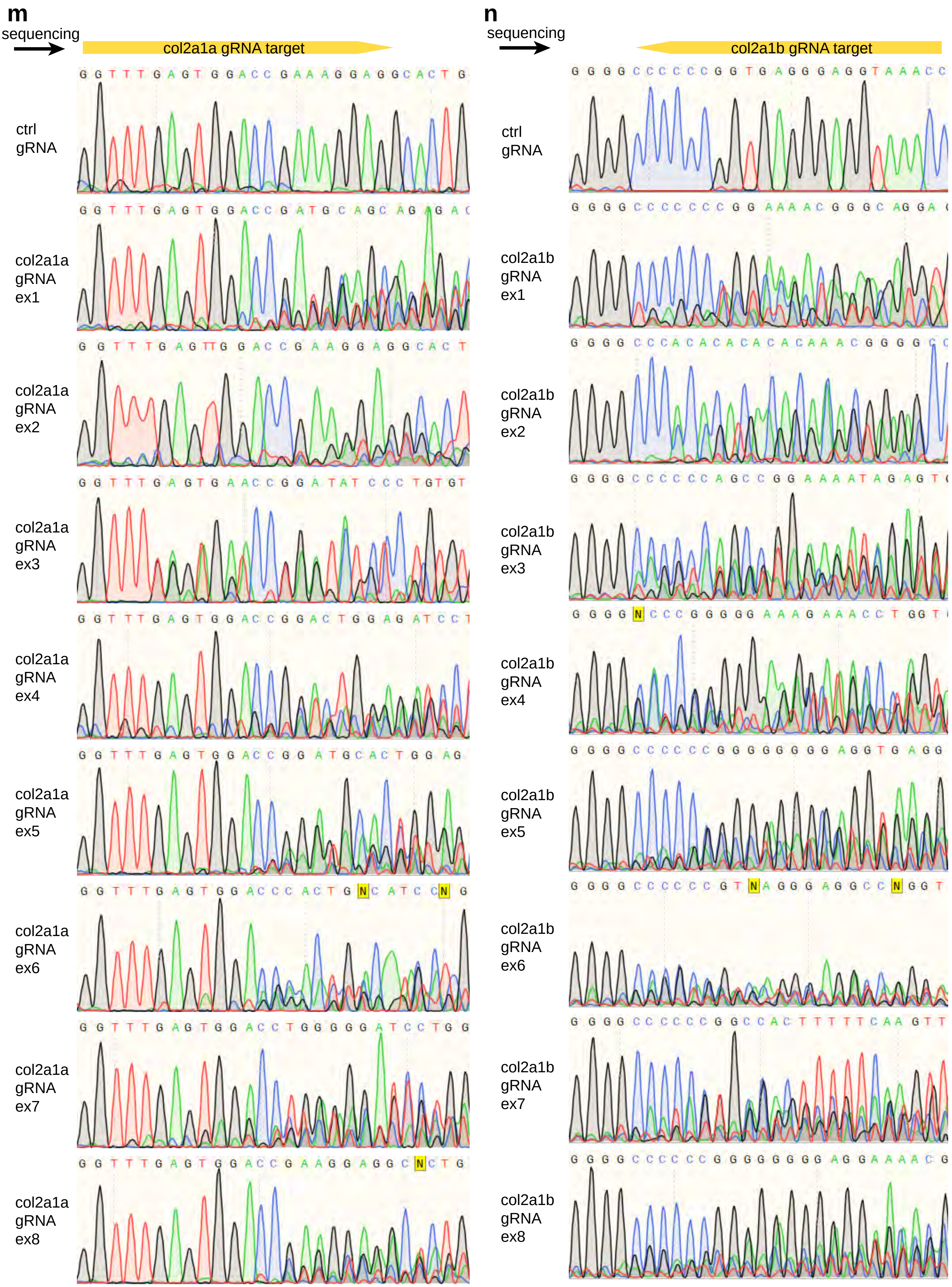
m
sequencing
col2a1a gRNA target
ctrl gRNA
col2a1a gRNA ex1
col2a1a gRNA ex2
col2a1a gRNA ex3
col2a1a gRNA ex4
col2a1a gRNA ex5
col2a1a gRNA ex6
col2a1a gRNA ex7
col2a1a gRNA ex8
n
sequencing
col2a1b gRNA target
ctrl gRNA
col2a1b gRNA ex1
col2a1b gRNA ex2
col2a1b gRNA ex3
col2a1b gRNA ex4
col2a1b gRNA ex5
col2a1b gRNA ex6
col2a1b gRNA ex7
col2a1b gRNA ex8

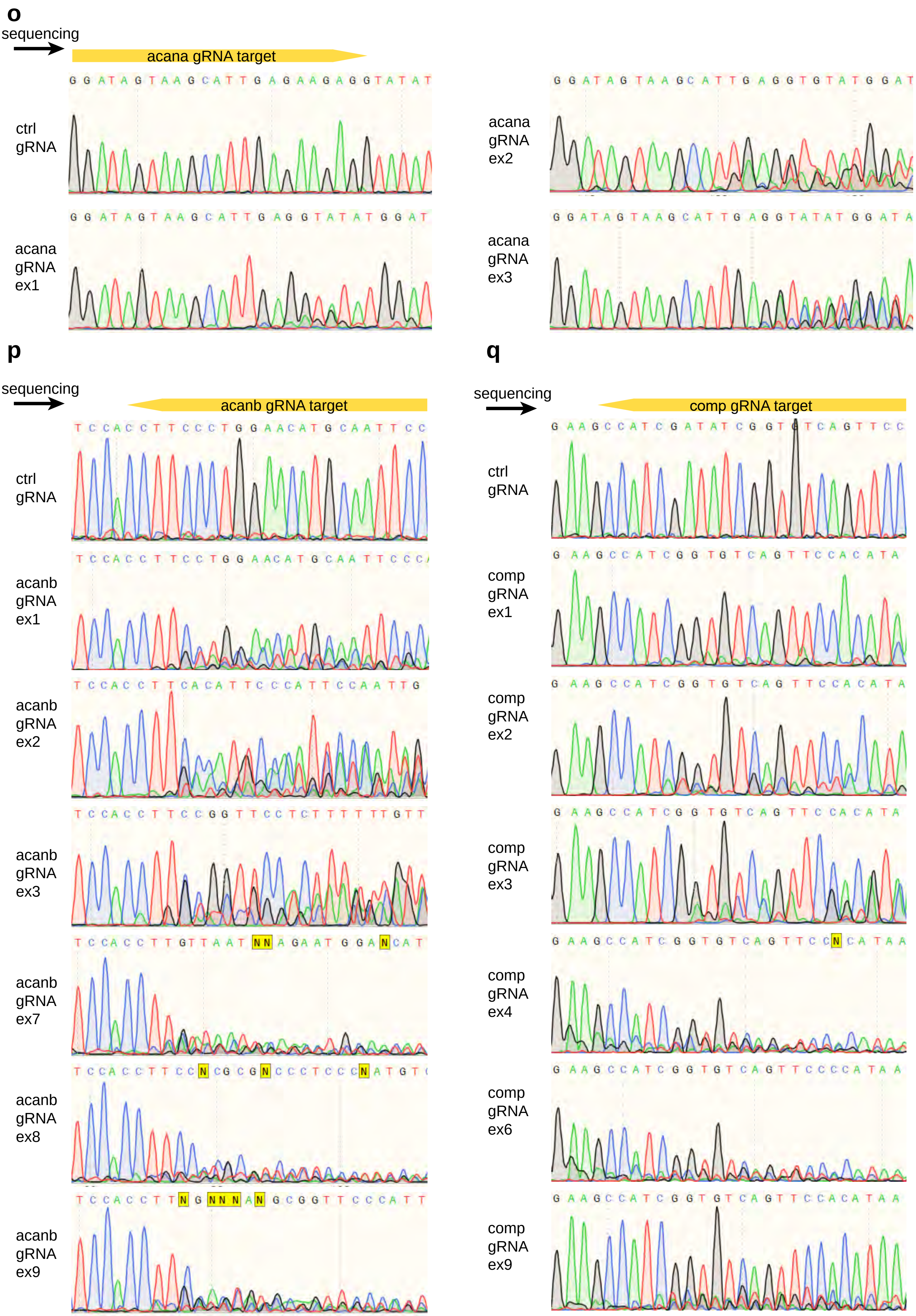
o
sequencing
acana gRNA target
ctrl gRNA
acana gRNA ex1
acana gRNA ex2
acana gRNA ex3
p
sequencing
acanb gRNA target
ctrl gRNA
acanb gRNA ex1
acanb gRNA ex2
acanb gRNA ex3
acanb gRNA ex7
acanb gRNA ex8
acanb gRNA ex9
q
sequencing
comp gRNA target
ctrl gRNA
comp gRNA ex1
comp gRNA ex2
comp gRNA ex3
comp gRNA ex4
comp gRNA ex6
comp gRNA ex9

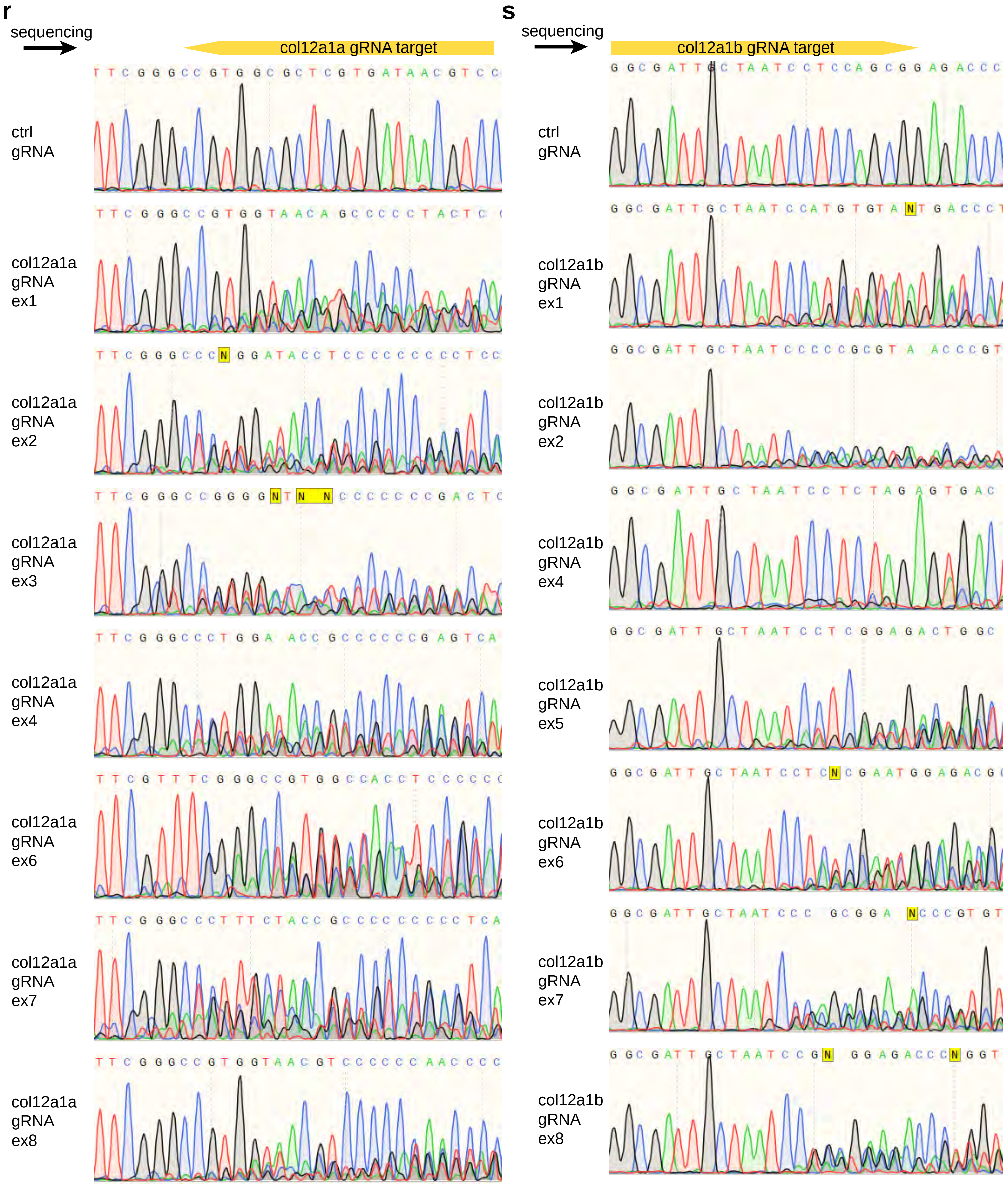
r
sequencing
col12a1a gRNA target
ctrl gRNA
col12a1a gRNA ex1
col12a1a gRNA ex2
col12a1a gRNA ex3
col12a1a gRNA ex4
col12a1a gRNA ex6
col12a1a gRNA ex7
col12a1a gRNA ex8
s
sequencing
col12a1b gRNA target
ctrl gRNA
col12a1b gRNA ex1
col12a1b gRNA ex2
col12a1b gRNA ex4
col12a1b gRNA ex5
col12a1b gRNA ex6
col12a1b gRNA ex7
col12a1b gRNA ex8

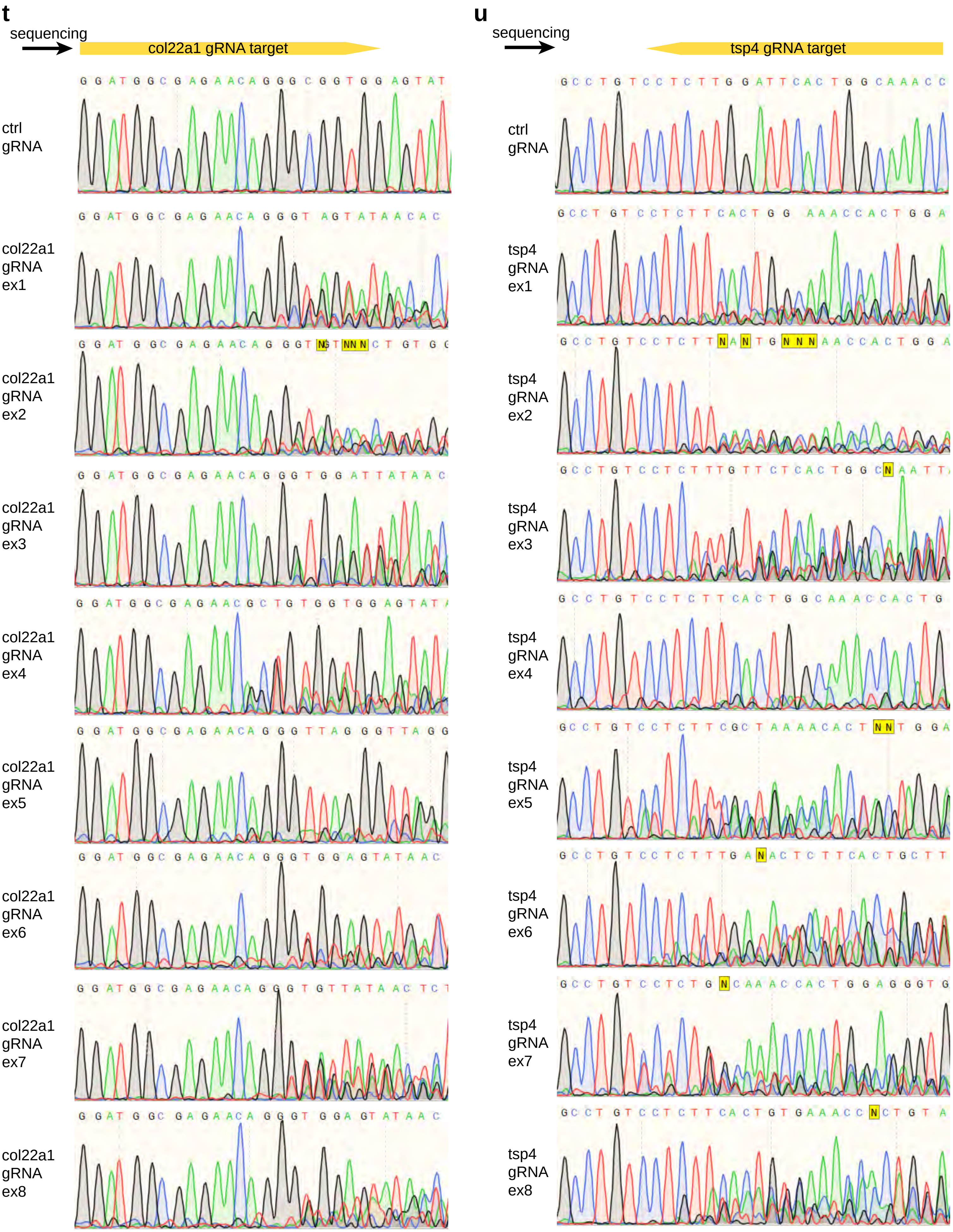
t
sequencing
col22a1 gRNA target
ctrl gRNA
col22a1 gRNA ex1
col22a1 gRNA ex2
col22a1 gRNA ex3
col22a1 gRNA ex4
col22a1 gRNA ex5
col22a1 gRNA ex6
col22a1 gRNA ex7
col22a1 gRNA ex8
u
sequencing
tsp4 gRNA target
ctrl gRNA
tsp4 gRNA ex1
tsp4 gRNA ex2
tsp4 gRNA ex3
tsp4 gRNA ex4
tsp4 gRNA ex5
tsp4 gRNA ex6
tsp4 gRNA ex7
tsp4 gRNA ex8

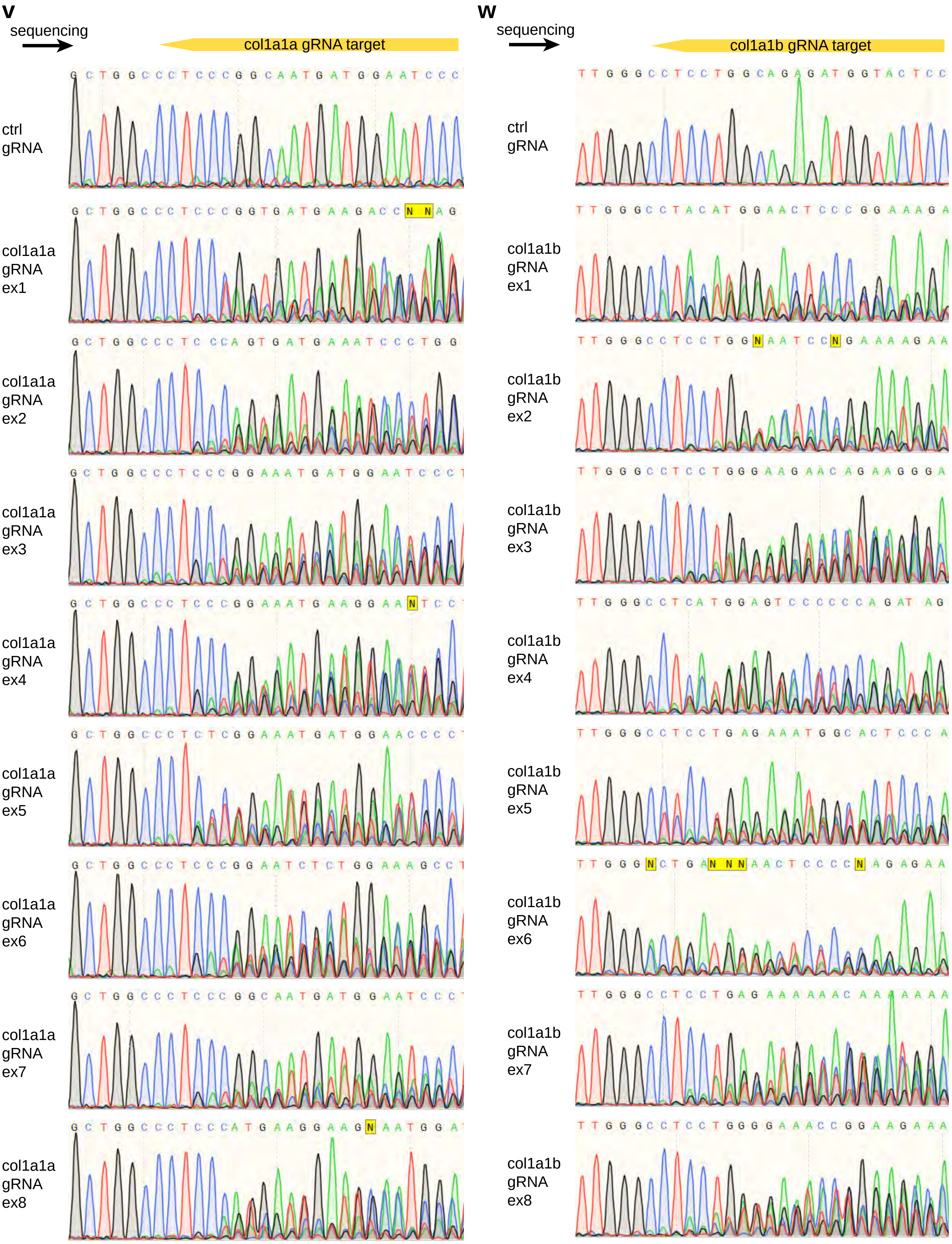
v
sequencing
col1a1a gRNA target
ctrl gRNA
col1a1a gRNA ex1
col1a1a gRNA ex2
col1a1a gRNA ex3
col1a1a gRNA ex4
col1a1a gRNA ex5
col1a1a gRNA ex6
col1a1a gRNA ex7
col1a1a gRNA ex8
w
sequencing
col1a1b gRNA target
ctrl gRNA
col1a1b gRNA ex1
col1a1b gRNA ex2
col1a1b gRNA ex3
col1a1b gRNA ex4
col1a1b gRNA ex5
col1a1b gRNA ex6
col1a1b gRNA ex7
col1a1b gRNA ex8

x

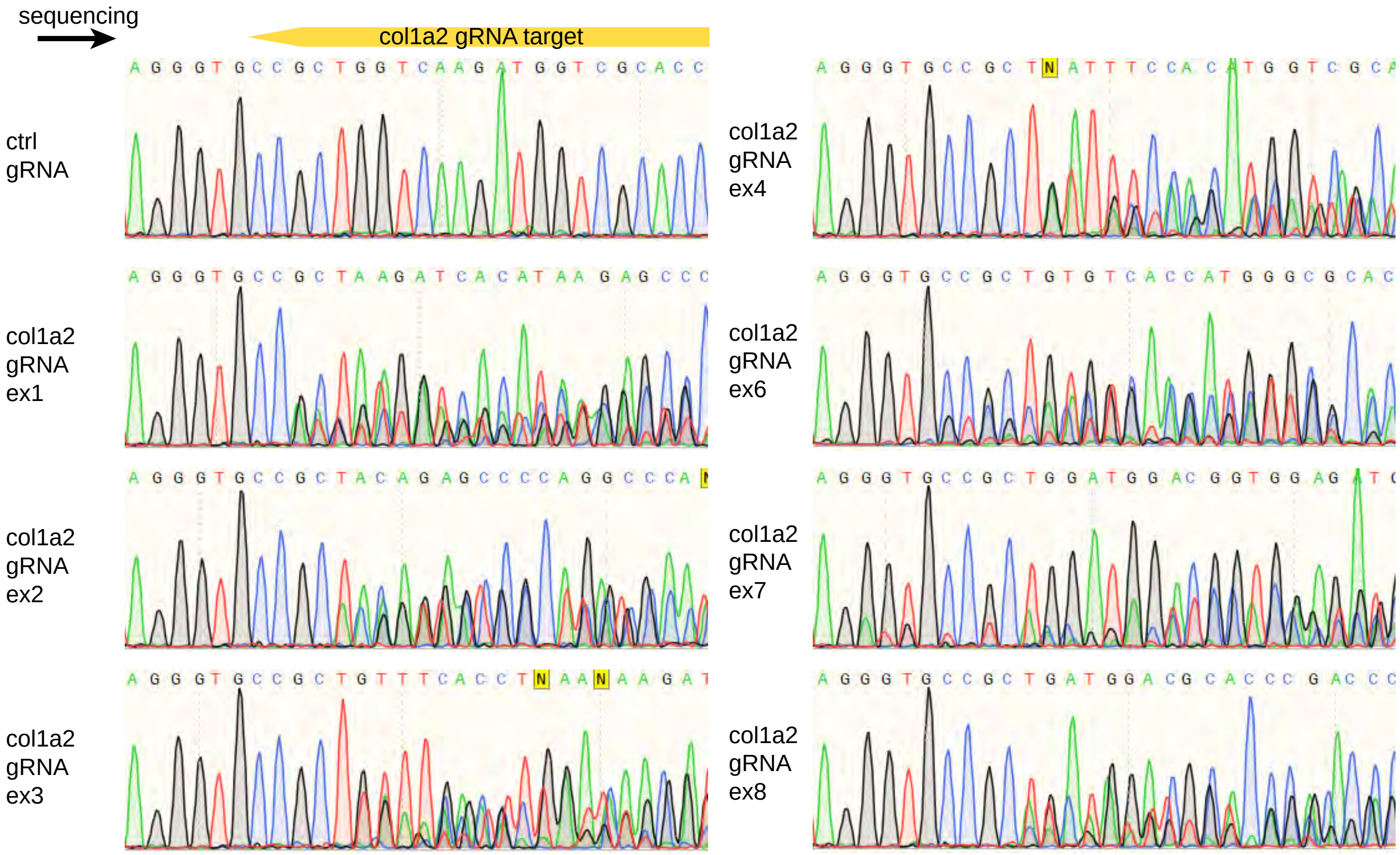
sequencing
col1a2 gRNA target
ctrl gRNA
col1a2 gRNA ex1
col1a2 gRNA ex2
col1a2 gRNA ex3
col1a2 gRNA ex4
col1a2 gRNA ex6
col1a2 gRNA ex7
col1a2 gRNA ex8

Supplementary Information

Sanger sequencing to assess CRISPR editing efficiency at targeted alleles. Results of sanger sequencing showing the targeted alleles of randomly selected embryos that were injected with the indicated gRNAs for all crispant embryo types used in the study. For each crispant, a control gRNA-injected embryo is shown above, and different examples for the crispant embryos display the variability of the editing state. Page 1: wnt5a (a), wnt5b (b). Page 2: wnt11 (c). Page 3: lamb1a (d), lamb1b (e), lambc1 (f). Page 4: sox9a (g), sox9b (h). Page 5: ttn.1 (i), ttn.2 (j). Page 6: itgb1a (k), itgb1b (l). Page 7: col2a1a (m), col2a1b (n). Page 8: acana (o), comp (q). Page 9: col12a1a (r), col12a1b (s). Page 10: col22a1 (t), tsp4 (u). Page 11: col1a1 (v), col1a1b (w). Page 12: col1a2 (x).
