# Supplementary material for: Directionality of developing skeletal muscles is set by mechanical forces
Source: Nat Commun. 2023 May 27;14:3060. doi: 10.1038/s41467-023-38647-7 (PMC10224984; doi:10.1038/s41467-023-38647-7)
Supplement: Supplementary file 3 — Description of Additional Supplementary Files [file 41467_2023_38647_MOESM3_ESM.pdf]

Description of Additional Supplementary Files

File Name: Supplementary Data 1
Description: DNA sequences

File Name: Supplementary Movie 1
Description: **Whole mount microscopy of musculoskeletal anatomy in zebrafish embryo.** The embryo of Tg(col2:mCherry) was stained with anti-DsRed (Col2) and antiMyHC antibody at 72 hpf. The facial region of the embryo was visualized with light-sheet microscopy. See also Fig. 1a.

File Name: Supplementary Movie 2
Description: **Extension of cartilage promotes the oriented extension of the attached muscle. The** embryo of Tg(col2:mCherry, tbx1:Cre) was injected with actb2:loxpmTagBFPcaax-loxp-EGFP and subjected to live imaging with light-sheet microscopy. Images were taken with 15 minutes interval for the indicated period. Animation was created with Fiji (7 frames per second). See also Fig. 1b.

File Name: Supplementary Movie 3
Description: **Slice view of musculoskeletal anatomy in control embryo.** The Tg(col2:mCherry) embryo was injected with control gRNA, and stained with anti-DsRed (Col2) and anti-MyHC antibody at 81 hpf. Animation was created with Fiji (7 frames per second). See also Fig. 1e.

File Name: Supplementary Movie 4
Description: **Slice view of musculoskeletal anatomy in Sox9- perturbed embryo**. The embryo of Tg(col2:mCherry) was injected gRNA targeting Sox9a/Sox9b, and was stained with anti-DsRed (Col2) and anti-MyHC antibody at 81 hpf. Animation was created with Fiji (7 frames per second). See also Fig.1e.

File Name: Supplementary Movie 5
Description: **Slice view of musculoskeletal anatomy in Runx2bperturbed embryo.** The embryo of Tg(col2:mCherry) was injected with morpholino targeting Runx2b, and stained with anti-DsRed (Col2) and anti-MyHC antibody at 81 hpf. Animation was created with Fiji (7 frames per second). See also Fig. 1e.

File Name: Supplementary Movie 6
Description: **Musculoskeletal anatomy of E11.5 mouse embryo by 3D reconstructions.** Localizations of Col2 and MyoD transcripts are visualized with whole mount RNA HCR in E11.5 wild-type embryo. See also Fig. 3a.

File Name: Supplementary Movie 7
Description: **Musculoskeletal anatomy of E12.5 mouse embryo by 3D reconstructions.** Localizations of Col2 and MyoD transcripts are visualized with whole mount RNA HCR in E12.5 wild-type embryo.

File Name: Supplementary Movie 8
Description: **Extension of the developing muscle from nonpolarized to polarized state.** The embryo from the outcross between Tg(actb2:loxp:sstop:loxp-DsRed) and Tg(fli:GFP, tbx1:Cre) were subjected to live imaging with light-sheet microscopy. Images were taken 20 minutes interval from 44 hpf to 72 hpf. DsRed is shown with green whereas GFP is shown with magenta. Animation was created with Fiji (9 frames per second). See also Fig. 4a.

File Name: Supplementary Movie 9
Description: **Genetic blockade of cartilage differentiation results in less-polarized muscle fibers.** The embryo from the outcross between Tg(actb2:loxp:sstop:loxp-DsRed) and Tg(fli:GFP, tbx1:CreERT2) was injected with gRNA targeting sox9a plus sox9b, or runx2b MO. Embryos were treated with 4OHT (10 µM) from 8 hpf to 39 hpf, and subjected to live imaging with light-sheet microscopy. Images of am muscle and pq cartilage were taken 15 minutes interval for the indicated time period. DsRed is shown with green whereas GFP is shown with magenta. 10-15 µm oblique slices were manually selected and exported in each time point, and animation was created with Fiji (7 frames per second). Non-injected embryos were served as control. See also Fig. 4b.

File Name: Supplementary Movie 10
Description: **Muscle polarization coincides with beginning of the interkinetic nuclear migration.** Embryos from the outcross between Tg(ubi:3905NLS, actb2:loxp:sstop:loxpDsRed) and Tg(tbx1:Cre) were subjected to live imaging with light-sheet microscopy. YFP (green) localized in nuclei from 3905NLS transgene is visualized only in DsRed-positive muscle lineage (red) using Imaris. Images of am muscle were taken 15 minutes interval from 48 hpf to 72 hpf. Animation was created with Fiji (7 frames per second).

File Name: Supplementary Movie 11
Description: **Laser ablation of the established attachment point results in viscoelastic contraction of the attached muscle cell.** The embryo from the outcross between Tg(unc503:gal4VP16) and Tg(col2:mCherry, UAS:GFP) was subjected to live imaging coupled with laser-ablation. Laser ablation was performed at 54 hpf and images were subsequently taken 0.5 sec interval for 45 sec. Animation was created with Fiji (7 frames per second). See Fig. 4g and 4h.

File Name: Supplementary Movie 12
Description: **Laser ablation of the prospective attachment points results in re-orienting muscles.** The embryo from the outcross between Tg(actb2:loxp:sstop:loxp-DsRed) and Tg(fli:GFP, tbx1:CreERT2) was treated with 4OHT (10 µM) from 8 hpf to 39 hpf, and then subjected to live-imaging coupled with laser-ablation. Laser ablation was performed at 42 hpf and images were subsequently taken 15 minutes interval for 6 hours. DsRed is shown with green whereas GFP is shown with magenta. Animation was created with Fiji (7 frames per second). See Fig. 4i.

File Name: Supplementary Movie 13
Description: **Tracking interkinetic nuclear migrations in developing muscles.** The embryo from the outcross between Tg(actb2:loxp:sstop:loxp-DsRed) and Tg(tbx1:CreERT2) were treated with 4OHT (10 µM) from 8 hpf to 48 hpf, and then subjected to live imaging with lightsheet microscopy. Images of cells in am muscle were taken 3 minutes interval from 50 hpf to 55 hpf. Animation was created with Fiji (14 frames per second). The moving nuclei were manually tracked with Imaris and the trajectory (XY plane, see also Fig. 3c) are shown in the end of the movie. See also Fig. 4j.

File Name: Supplementary Movie 14
Description: **Orientation of myocyte cell division is not disrupted in the absence of cartilage attachment.** The embryo from the outcross between Tg(actb2:loxp:sstop:loxp-DsRed) and Tg(fli:GFP, tbx1:CreERT2) were injected with gRNA targeting sox9a plus sox9b, or runx2b MO. Embryos were treated with 4OHT (10 µM) from 8 hpf to 48 hpf, and subjected to live imaging with light-sheet microscopy. Images of individual cells in am muscle were taken 5 minutes interval from 48 hpf to 58 hpf to find dividing cells. 3 µm oblique slices were manually selected and exported in each time point, and

animation was created with Fiji (10 frames per second). Non-injected embryos served as control. See also Fig. 4k.

File Name: Supplementary Movie 15
Description: **Synchronization of myocyte orientation on a continuously stretched membrane.** C2C12 cells were seeded on laminincoated PDMS membrane, and transfected with GFP. Cells were induced to differentiate and then immediately stretched 20%. Images of non-stretched and stretched cells were taken from 1 to 51 hours of differentiation with 15 minutes interval. Animation was created with Fiji (20 frames per second). See also Fig. 6d-g.

File Name: Supplementary Movie 16
Description: **Defective oriented extension of myofibers in the laminin-deficient zebrafish embryos.** Embryos from the outcross between Tg(actb2:loxp:sstop:loxp-DsRed) and Tg(fli:GFP, tbx1:CreERT2) were injected with gRNA targeting lamc1. The embryos were then treated with 4OHT (10 µM) from 8 hpf to 39 hpf, and subjected to live imaging with light-sheet microscopy. Images of cells in am muscle and pq cartilage were taken 15 minutes interval for the indicated time period. DsRed is shown with green whereas GFP is shown with magenta. 18 µm oblique slices were manually selected and exported in each time point, and animation was created with Fiji (7 frames per second). See also Supplementary Fig. 10f.

File Name: Supplementary Movie 17
Description: **Defective orientation of myocytes in the presence of JNK inhibitors.** Embryos from the outcross between Tg(actb2:loxp:sstop:loxp-DsRed) and Tg(fli:GFP, tbx1:CreERT2) were treated with 4OHT (10 µM) from 8 hpf to 39 hpf. The embryos were treated with 20 µM SP600125 and immediately subjected to time-lapse imaging. Images of cells in am muscle and pq cartilage were taken 15 minutes interval for the indicated time period. 10 or 20 µm oblique slices were manually selected for control (nontreated) or SP600125-treated embryo, and exported in each time point. The animation was created with Fiji (7 frames per second). See also Fig. 7i.

File Name: Supplementary Movie 18
Description: **Trunk myocytes elongate without attaching to cartilage.** AB embryo was injected with myog:EGFP-caax-p2A-h2afv-mCherry and subjected to live imaging with confocal microscopy. Images of trunk muscle were taken 15 minutes interval from 21 hpf to 38 hpf. Animation was created with Fiji (7 frames per second).
