# Supplementary material for: Directionality of developing skeletal muscles is set by mechanical forces
Source: Nat Commun. 2023 May 27;14:3060. doi: 10.1038/s41467-023-38647-7 (PMC10224984; doi:10.1038/s41467-023-38647-7)
Supplement: Supplementary file 23 — Reporting Summary [file 41467_2023_38647_MOESM23_ESM.pdf]

nature portfolio

Corresponding author(s): Igor Adameyko

Last updated by author(s): May 3, 2023

Reporting Summary

Nature Portfolio wishes to improve the reproducibility of the work that we publish. This form provides structure for consistency and transparency in reporting. For further information on Nature Portfolio policies, see our Editorial Policies and the Editorial Policy Checklist.

Statistics

For all statistical analyses, confirm that the following items are present in the figure legend, table legend, main text, or Methods section.

n/a	Confirmed	
☐	☒	The exact sample size (*n*) for each experimental group/condition, given as a discrete number and unit of measurement
☐	☒	A statement on whether measurements were taken from distinct samples or whether the same sample was measured repeatedly
☐	☒	The statistical test(s) used AND whether they are one- or two-sided<br>*Only common tests should be described solely by name; describe more complex techniques in the Methods section.*
☒	☐	A description of all covariates tested
☐	☒	A description of any assumptions or corrections, such as tests of normality and adjustment for multiple comparisons
☐	☒	A full description of the statistical parameters including central tendency (e.g. means) or other basic estimates (e.g. regression coefficient) AND variation (e.g. standard deviation) or associated estimates of uncertainty (e.g. confidence intervals)
☐	☒	For null hypothesis testing, the test statistic (e.g. *F*, *t*, *r*) with confidence intervals, effect sizes, degrees of freedom and *P* value noted<br>*Give P values as exact values whenever suitable.*
☒	☐	For Bayesian analysis, information on the choice of priors and Markov chain Monte Carlo settings
☒	☐	For hierarchical and complex designs, identification of the appropriate level for tests and full reporting of outcomes
☐	☒	Estimates of effect sizes (e.g. Cohen's *d*, Pearson's *r*), indicating how they were calculated

Our web collection on statistics for biologists contains articles on many of the points above.

Software and code

Policy information about availability of computer code

Data collection	Zen Blue and Zen Black (Zeiss), IMSpector TM347, ClickPoints (Gerum), GE phoenix datos|x (GE Sensing and Inspection Technologies), VG Studio Max 3.2 (GMBH), Avizo (ThermoFisher)
Data analysis	ImageJ/FIJI, Imaris File Converter and Imaris (Bitplane version 9.2.1), Zen Blue and Zen Black (Zeiss) are used for image analysis. R (4.1.3) and Python (3.8.14) were used to analyze transcriptomics datasets. To access the code used for analysis of transcriptomics data, please see the following Github: https://github.com/LouisFaure/muscledirection_paper (DOI:10.5281/zenodo.7792602)

For manuscripts utilizing custom algorithms or software that are central to the research but not yet described in published literature, software must be made available to editors and reviewers. We strongly encourage code deposition in a community repository (e.g. GitHub). See the Nature Portfolio guidelines for submitting code & software for further information.

Data

Policy information about availability of data

All manuscripts must include a data availability statement. This statement should provide the following information, where applicable:

- Accession codes, unique identifiers, or web links for publicly available datasets
- A description of any restrictions on data availability
- For clinical datasets or third party data, please ensure that the statement adheres to our policy

All data required to assess the claims of this study are included in the manuscript. Any additional datasets generated during and/or analyzed during the current study are available from the corresponding author on reasonable request. Transcriptomics datasets generated during this study have been deposited to GEO

database under accession codes GSE160098 (single cell RNA sequencing) and GSE199093 (bulk RNA sequencing).
To access the single-cell transcriptomics dataset used in this study, please use the following link:
https://www.ncbi.nlm.nih.gov/geo/query/acc.cgi?acc=GSE160098
The bulk RNA sequencing data can be accessed here:
https://www.ncbi.nlm.nih.gov/geo/query/acc.cgi?acc=GSE199093

Research involving human participants, their data, or biological material

Policy information about studies with human participants or human data. See also policy information about sex, gender (identity/presentation), and sexual orientation and race, ethnicity and racism.

Reporting on sex and gender	The study analyzes the development of zebrafish and mouse embryos only. We did not collect the information about sex of the embryos, as this study focuses on early developmental events that should not be affected significantly by sexual dimorphism.
Reporting on race, ethnicity, or other socially relevant groupings	The study has no human participants.
Population characteristics	See above.
Recruitment	See above.
Ethics oversight	See above.

Note that full information on the approval of the study protocol must also be provided in the manuscript.

Field-specific reporting

Please select the one below that is the best fit for your research. If you are not sure, read the appropriate sections before making your selection.

[x] Life sciences [ ] Behavioural & social sciences [ ] Ecological, evolutionary & environmental sciences

For a reference copy of the document with all sections, see nature.com/documents/nr-reporting-summary-flat.pdf

Life sciences study design

All studies must disclose on these points even when the disclosure is negative.

Sample size

Sample sizes were not statistically calculated prior to the study. Generally, with comparisons of individual embryos we measured 3-10 embryos per condition. When comparing individual cells we measured dozens and up to hundreds of cells per condition. In all cases we acquired data for each condition until the distribution was evident. The sample sizes are large enough to statistically differentiate two groups that have evidently different distributions.

Data exclusions

Col2creERT;R26DTA system is used for a mouse model of cartilage ablation to test effects on muscle shape. Rarely, recombination of DTA is inefficient and cartilage grows normally. We excluded one such DTA embryo from the analysis, as cartilage-ablation was the prerequisite for the comparison. For the single-cell transcriptomic analysis, we excluded single cells from the analysis that had fewer than 1000 genes.

Replication

Major experiments were performed twice, with similar results, except when otherwise specified. When analyzing muscles in the zebrafish embryo, we analyzed multiple biologically independent replicate embryos, except when otherwise specified. Col2creERT;R26DTA embryos were from a single litter. When analyzing cells in culture, we analyzed multiple independent cultures. All replication attempts have been successful. For panels where statistical significance is determined, all information about replication is contained within the source data file.

Biological N values for micrographs in Main Figures are as follows:
1a, n > 3 embryos across n=3 experiments
1b, n = 1 embryos across n = 1 experiments
1c, n = 6 embryos for each control or mutant across n=3 experiments
1h, n = 3 embryos for ctrl, n = 4 for sox9a+b, n = 3 for runx2b, across n= 2 experiments
2f, n = 7 embryos for ctrl, n = 8 for sox9a+b, n = 7 for runx2b, across n = 2 experiments
2g, n=3 embryos for 48h, n = 4 for 52h, n = 4 for 81h, across n=2 experiments
2h, n = 3 embryos for ctrl, n = 3 for sox9a/b gRNA, n = 2 for runx2b MO, across n=1 experiments
2i: n = 3 embryos for ctrl, n = 3 for sox9, n = 3 for runx2b, across n=2 experiments
3a: n = 2 embryo, across n=2 experiments
3b: n = 2 embryo, across n=2 experiments
3g-i: n = 1 embryo, across n=1 experiment
6m: n = 4 devices across n=2 experiments
6n: n = 3 devices across n=2 experiments

6o: n = 3 devices across n=1 experiments
6p: n = 4 devices across n=1 experiments
7a,b: n =2 for control, n = 2 for SP600125, across n=2 experiments
7g, h: n = 2 for control, n = 4 for SP600125, across n=1 experiment
7i: n = 1 embryo across n=1 experiment

Biological N values for micrographs in Supplementary Figures are as follows:
1a, b: n >= 3 embryos, across n>=3 experiments
1c: n = 4 embryos for WT, n = 4 for Sox9a/Sox9b gRNA, across n=1 experiment
1d-e: n = 3 embryos, across n=1 experiment
2a: n = 3 embryos, across n=1 experiment
2b: n = 2 embryos for control gRNA, n = 3 for Tsp4b, Col12a1a, Col12a1b, Col22a1a, gRNA across n=1 experiment
2c: n = 3 embryos for control gRNA, n = 5 for Col1a1a, Col1a1b, Col1a2 gRNA, across n=1 experiment
3a:n = 3 embryos, n=2 experiments
3b:n = 2 embryos, across n=1 experiment
3c,d: n = 3 embryos across n= 1 experiment
3e-s: n = 1 embryos across n=1 experiment
5a-c: n = 5 for ctrl, n = 5 for sox9a+9b gRNA, n = 5 for runx2b MO across n=1 experiment
6a-d: n = 3 embryos, across n=1 experiment
8a: n = 3, across n=3 experiments
9a: n = 2 for itgb1a, n = 2 for itgb1b, across n=1 experiment
9f: n = 3 for Ctrl, n = 5 for itgb1a + b gRNA, across n=2 experiments
10a: n = 4 across n=2 experiments
10b: n = 4 for control, n = 4 for sox9a + 9b gRNA, n = 3 for runx2b across n=1 experiment
10c: n = 4 for lamb1a, n = 4 for lamb1b, n = 4 for lamc1 across n=1 experiment
10e: n = 5 for ctrl gRNA, n = 8 for ECM gRNA, n = 9 for lamb1 gRNA, n = 8 for lamc1 gRNA across n=2 experiments
10f: n = 1 across n=1 experiment

Randomization

Samples were randomly allocated into experimental groups. For some quantifications of myocyte length and fusion, the same control group sample was used for different experiments (this is detailed in the excel spreadsheet showing all data values)

Blinding

Blinding was not performed during analysis or collection, because the differences being analyzed (muscle phenotype) are secondary to primary differences (cartilage growth defect). Knowing this, blinding would not be feasible, as samples look very different in known ways. Samples were treated equivalently in the analysis.

Reporting for specific materials, systems and methods

We require information from authors about some types of materials, experimental systems and methods used in many studies. Here, indicate whether each material, system or method listed is relevant to your study. If you are not sure if a list item applies to your research, read the appropriate section before selecting a response.

Materials & experimental systems

n/a	Involved in the study	
☐	☒	Antibodies
☐	☒	Eukaryotic cell lines
☒	☐	Palaeontology and archaeology
☐	☒	Animals and other organisms
☒	☐	Clinical data
☒	☐	Dual use research of concern
☒	☐	Plants

Methods

n/a	Involved in the study	
☒	☐	ChIP-seq
☒	☐	Flow cytometry
☒	☐	MRI-based neuroimaging

Antibodies

Antibodies used

anti-MyHC antibody (1:40, A4.1025, DSHB)
anti-MyHC antibody (1:200, F59, DSHB)
anti-DsRed antibody (1:200, 632496, Clontech)
anti-Laminin antibody (1:100, L9393, Sigma)
anti-GFP antibody (1:200, ab6662, Abcam)
anti-Fluor-POD (Sigma, 1:5000)
anti-DIG-POD (Sigma, 1:2000)
anti-DNP-POD (Perkin Elmer, 1:500)
Alexa Fluor 647 donkey anti-mouse IgG (H+L) (1:500, A31571, Thermo Fisher Scientific)

	Alexa Fluor 555 donkey anti-rabbit IgG (H+L) (1:500, A31572, Thermo Fisher Scientific)

Validation

anti-MyHC antibody (1:40, A4.1025, DSHB). Mouse monoclonal. Immunogen: Myosin heavy chain (human MHC or MyHC) purified. Reactivity: Danio rerio (Zebrafish) Homo sapiens (Human) Rattus norvegicus (Rat)

anti-MyHC antibody (1:200, F59, DSHB). Mouse monoclonal. Immunogen: Myosin isolated from pectoralis major. Species Reactivity: Amphibian, Avian, Canine, Fish, Human, Mouse, Quail, Rabbit, Rat, Shark, Turtle, Xenopus, Zebrafish. Depositors Notes: This antibody is specific for myosin heavy chain in fast contracting skeletal muscle and atrial and ventricular myosin heavy chains.

anti-DsRed antibody (1:200, 632496, Clontech). The Living Colors DsRed Polyclonal Antibody has been raised against DsRed-Express, a variant of Discosoma sp. red fluorescent protein. This antibody recognizes DsRed-Express, DsRed-Express2, DsRed-Monomer, mCherry, DsRed2, E2-Crimson, tdTomato, mStrawberry, and mBanana, and both N- and C-terminal fusion proteins containing these fluorescent proteins in mammalian cell lysates. 100ul

anti-Laminin antibody (1:100, L9393, Sigma). Rabbit polyclonal. Immunogen: Laminin isolated from the basement membrane of Englebreth Holm-Swarm (EHS) mouse sarcoma. Species reactivity, animal, human. A working dilution of at least 1:1,000 was determined by a dot blot immunoassay using laminin at 50 ng per dot. A working dilution of at least 1:25 was determined by indirect immunohistology using formalin-fixed, paraffin-embedded human and animal tissues.

anti-GFP antibody (1:200, ab6662, Abcam). Goat polyclonal. Immunogen: Recombinant full length protein corresponding to GFP aa 1-246. Binds to similar related fluorescent proteins, YFP, CFP, etc.

anti-Fluor-POD (Sigma, 1:5000). Sheep polyclonal. Fab fragments from polyclonal anti-fluorescein antibodies, conjugated to horseradish peroxidase. The polyclonal antibody reacts with free and bound fluorescein. Dot blot: 150 mU/ml ELISA: 50 to 150 mU/ml Immunohistocytochemistry: 250 to 500 mU/ml In situ hybridization: 1.5 to 7.5 U/ml Southern blot: 150 mU/ml Western blot: 500 to 1000 mU/ml

anti-DIG-POD (Sigma, 1:2000). The polyclonal antibody from sheep is specific to digoxigenin and digoxin and shows no cross-reactivity with other steroids, such as human estrogens and androgens.

anti-DNP-POD (Perkin Elmer, 1:500). Part of TSA DNP-HRP kit.

Alexa Fluor 647 donkey anti-mouse IgG (H+L) (1:500, A31571, Thermo Fisher Scientific). Cross-adsorbed against bovine, chicken, goat, guinea pig, hamster, horse, human, rabbit, rat, and sheep serum. Anti-Mouse secondary antibodies are affinity-purified antibodies with well-characterized specificity for mouse immunoglobulins and are useful in the detection, sorting or purification of its specified target.

Alexa Fluor 555 donkey anti-rabbit IgG (H+L) (1:500, A31572, Thermo Fisher Scientific). Cross-adsorbed against bovine, chicken, goat, guinea pig, hamster, horse, human, mouse, rat, and sheep serum. Anti-Rabbit secondary antibodies are affinity-purified antibodies with well-characterized specificity for rabbit immunoglobulins and are useful in the detection, sorting or purification of its specified target

Eukaryotic cell lines

Policy information about cell lines and Sex and Gender in Research

Cell line source(s)	C2C12 were purchased from ATCC
Authentication	C2C12 were validated by their morphology and tendency to form myotubes.
Mycoplasma contamination	Mycoplasma was not detected in the cells after purchase from the ATCC company, and cells were not regularly tested for mycoplasma thereafter.
Commonly misidentified lines (See ICLAC register)	The study does not make use of commonly misidentified cell lines.

Animals and other research organisms

Policy information about studies involving animals; ARRIVE guidelines recommended for reporting animal research, and Sex and Gender in Research

Laboratory animals

Zebrafish and mice were used. Both sexes were used for breeding and for the experiments. The ages analyzed: fish, between 1-5 dpf. mouse, between 11 and 17 dpc. Zebrafish were raised in E3 medium (5 mM NaCl, 0.17 mM KCl, 0.33 mM $CaCl_2$, 0.33 mM $MgSO_4$, and 2 mM HEPES) at 28.5°C.

Col2a1-CreERT2 strains [90] were obtained from the laboratory of S. Mackem, NIH. DTA strain [91] (B6.129P2-Gt(ROSA)26Sortm1

(DTA)Lky/J, The Jackson Laboratory) was coupled to Col2a1-CreERT2. Mice were kept in an SPF animal facility with standardized conditions (24oC, 12:12 h light–dark cycle, normal humidity, food and water ad libitum).

Tg(col2:mCherry), Tg(fli:GFP), and Tg(actb2:loxp:sstop:loxp-DsRed) are described previously (Lawson and Weinstein, 2002; Mitchell et al., 2013: Bertrand et al., 2010). Tg(tbx1:Cre) and Tg(tbx1:CreERT2) were generated with the Tol2 transposon/transposase system[80, 81] as follows: the region between -5717 bp and -7 bp from the start codon of tbx1 gene was cloned to p5E vector with Gateway BP Clonase II (Thermo Fisher Scientific). Also, Cre recombinase of pCS2-Cre.zf1 (Addgene plasmid #61391) was transferred to pME vector by BP reaction. The expression construct was generated by recombining p5E-tbx1, pME-Cre (or CreERT2, Addgene plasmid #27321), p3E-polyA (tol2 kit #302) and pDestTol2CG2 (tol2 kit #395) with Gateway LR Clonase II (Thermo Fisher Scientific). Similarly, Tg(ubi:3905NLS) was generated by recombining p5E-ubi, pME-3905NLS, p3E-poly and pDestTol2pA2. pME-3905NLS was created by transferring the insert of pT2A-3905NLS [82] to the pME vector. Transposase RNA was prepared using the pCS2FA-transposase as a template as described previously[81]. Briefly, in vitro transcription was performed using mMessage mMachine SP6 kit (Thermo Fisher Scientific), and RNA was purified with RNeasy Mini Kit (Qiagen). 24 ng expression construct was injected with 20 ng Transposase RNA to AB line, and embryos were sorted according to fluorescence. Then obtained founder fishes were outcrossed with AB line. For transient expression of Cre-inducible GFP cassette, actb2:loxp-mTagBFPcaax-loxp-EGFP was created as follows: the 10,175 bp promoter of Actb2 (Bertrand et al., 2010) was cloned to the p5E vector by BP reaction. Then loxp-mTagBFPcaax-loxp sequence was inserted after Actb2 promoter from pENTR5'_ubi:loxP-EGFP-loxP (Addgene plasmid #27322) and pME-mTagBFPcaax (Addgene plasmid #75149) with restriction enzyme reactions. The expression vector was then created by the recombination of p5E-actb2:loxp-mTagBFPcaax-loxp, pME-EGFP (tol2 kit #383), p3E-polyA, and pDestTol2CG2. For the generation of myog:DVLDEP-P2A-EGFP, the promoter region of zebrafish myogenin (-956 bp – 0 bp) was cloned to p5E vector, and human DVL3 lacking DEP region was cloned to pME-p2A-EGFP vector, and then they were recombined to pDestTol2CG2. For the generation of unc503:Gal4VP16, p5E_unc503 (Addgene plasmid # 64020), pME-Gal4VP16 (tol2 kit, #387), and p3E-polyA were recombined to pDESTtol2pACrymCherry (Addgene plasmid # 64023). Primer sequences used for the promoter cloning are shown in Supplementary Table 1. Stable mutant lines for gene knockout, Sox9atw37(item #348), Runx2bsa14504 (item#14978), Sox9bsa40193 (item#36119), and Itgba1sa8902 (item#37693) were purchased from the European Zebrafish Resource Center.

Wild animals	No wild animals were used.
Reporting on sex	The findings are important for both sexes equally.
Field-collected samples	No field collection.
Ethics oversight	All experimental work with animals was permitted by the Ethical Committee on Animal Experiments (Stockholm North committee) and conducted according to The Swedish Animal Agency's Provisions and Guidelines for Animal Experimentation recommendations.

Note that full information on the approval of the study protocol must also be provided in the manuscript.
